# Positive strand RNA viruses differ in the constraints they place on the folding of their negative strand

**DOI:** 10.1261/rna.079125.122

**Published:** 2022-10

**Authors:** Morgan R. Herod, Joseph C. Ward, Andrew Tuplin, Mark Harris, Nicola J. Stonehouse, Christopher J. McCormick

**Affiliations:** 1 School of Molecular and Cellular Biology, Faculty of Biological Sciences and Astbury Centre for Structural Molecular Biology, University of Leeds, Leeds LS2 9JT, United Kingdom; 2 Clinical and Experimental Sciences, Faculty of Medicine, University of Southampton, Sir Henry Wellcome Laboratories, University Hospital Southampton, Southampton SO16 6YD, United Kingdom; 3 Institute for Life Sciences, University of Southampton SO17 1BJ, United Kingdom

**Keywords:** positive strand RNA virus, replication, replication intermediate, ribozyme, double-stranded RNA

## Abstract

Genome replication of positive strand RNA viruses requires the production of a complementary negative strand RNA that serves as a template for synthesis of more positive strand progeny. Structural RNA elements are important for genome replication, but while they are readily observed in the positive strand, evidence of their existence in the negative strand is more limited. We hypothesized that this was due to viruses differing in their capacity to allow this latter RNA to adopt structural folds. To investigate this, ribozymes were introduced into the negative strand of different viral constructs; the expectation being that if RNA folding occurred, negative strand cleavage and suppression of replication would be seen. Indeed, this was what happened with hepatitis C virus (HCV) and feline calicivirus (FCV) constructs. However, little or no impact was observed for chikungunya virus (CHIKV), human rhinovirus (HRV), hepatitis E virus (HEV), and yellow fever virus (YFV) constructs. Reduced cleavage in the negative strand proved to be due to duplex formation with the positive strand. Interestingly, ribozyme-containing RNAs also remained intact when produced in vitro by the HCV polymerase, again due to duplex formation. Overall, our results show that there are important differences in the conformational constraints imposed on the folding of the negative strand between different positive strand RNA viruses.

## INTRODUCTION

Positive strand RNA viruses infect all forms of life and impose significant environmental, economic and social hardship due to the diseases that they cause. For those viruses that infect higher eukaryotic organisms, viral genome replication occurs in membrane rich compartments within the cytosol often referred to as replication complexes (RCs) (den Boon and Ahlquist 2010). These compartments are thought to concentrate the viral and host proteins needed for genome replication (den Boon and Ahlquist 2010; Li and Nagy 2011) as well as protect the viral RNAs produced in them from intrinsic immune sensors and the action of antiviral proteins (Uchida et al. 2014; Kovalev et al. 2017).

The basic principles of viral genome replication were first determined by pulse chase experiments that allowed RNA synthesis to be followed over time (Montagnier and Sanders 1963; Baltimore et al. 1964; Brown and Cartwright 1964; Friedman et al. 1966; Baltimore 1968; Cleaves et al. 1981; Chu and Westaway 1985). The current paradigm, based on these studies, is that genomic RNA ([+] RNA) is first recruited to the RC before serving as a template for production of a complementary negative strand RNA ([−] RNA) intermediate. This [−] RNA is closely associated with the [+] RNA from which it was synthesized, forming a double-stranded RNA (dsRNA) complex on extraction from cells that is referred to as the replicative form (RF) (Montagnier and Sanders 1963; Baltimore et al. 1964). The [−] RNA within the RF subsequently serves as a template for [+] RNA synthesis. Production of [−] and [+] RNA is asymmetric, with multiple copies of the [+] RNA at various stages of synthesis typically being found associated with a single [−] RNA template. In this state, the RNA species present is known as a replicative intermediate (RI) and when extracted from cells appears as a double-stranded structure with multiple single-stranded branches extending from it, with each branch representing a partially synthesized nascent [+] RNA (Brown and Cartwright 1964; Friedman et al. 1966; Baltimore 1968; Cleaves et al. 1981; Chu and Westaway 1985). Although formal demonstration of RF and RI formation has typically been restricted to those viruses that show robust replication, this process is believed to be the same for all positive strand RNA viruses infecting higher eukaryotic organisms but adapted or extended for those viruses producing subgenomic RNAs (sgRNAs) in addition to their full length genome.

Intramolecular RNA structures encoded within the viral genome play a key role in the viral replication cycle. Many viruses use an internal ribosome entry site (IRESes) as a substitute for a 5′ cap to ensure translation of their viral proteins (Jang et al. 1988; Pelletier and Sonenberg 1988). Use of stem–loops within coding regions to control frameshifting is also common (Brierley et al. 1989; ten Dam et al. 1990; Chung et al. 2010). Importantly, RNA structure is an integral part of many *cis*-acting replication elements (CREs); RNA elements that play a more direct role in genome replication itself. The function of these CRE elements ranges from controlling the switch between translation to replication (Gamarnik and Andino 1998; Tuplin et al. 2015; Liu et al. 2016; Sanford et al. 2019), acting as cofactors in enzymatic processes such as viral protein uridylation (Paul et al. 2000) and serving as promoters to direct initiation of viral polymerase activity (Song and Simon 1995; Olsthoorn and Bol 2002; Filomatori et al. 2006; Yunus et al. 2015).

The single-stranded nature of the viral genome provides the CREs within it the relative freedom to fold. However, once [−] RNA synthesis has occurred, constraints are encountered. This is because the complementary nature of the [−] and [+] RNAs promotes dsRNA formation. Despite this several viruses harbor structured CREs in their [−] RNA; CREs that act as promoters for genomic and subgenomic RNA production (Grdzelishvili et al. 2000; Li et al. 2002; Friebe and Bartenschlager 2009; Yunus et al. 2015; Schult et al. 2019). Thus, at some point the duplex base pairing masking these CREs has to be separated in such a way that intramolecular base pairing is promoted while intermolecular base pairing is prevented. To date, the only way used to monitor RNA folding in the [−] RNA has been to use CRE-dependent replication as a readout (Friebe and Bartenschlager 2009; Yunus et al. 2015; Schult et al. 2019). As CRE function is specific to the virus, this does not offer a uniform way to examine RNA folding across the [−] RNA of different viruses. Indeed, to our knowledge, the issue of whether sequences other than CRE elements are able to adopt an RNA fold within the [−] strand has never been addressed. Part of the reason is that many RNA structural analysis techniques require disruption of the RC, an action which facilitates the collapse of RF and RI forms into a double-stranded state (Richards et al. 1984; Fujimura et al. 2005). Furthermore, the few techniques that allow RNA structure to be examined in situ (Richards et al. 1984; Spitale et al. 2013) are hampered by the typical low abundance of the RF and RI in the infected cell. Thus, any technique that offers the ability to monitor RNA folding in situ, and to be able to extend this analysis to structures beyond the confines of CRE elements, has the potential to provide valuable insight into the inner workings of the viral RC.

Ribozymes are self-cleaving RNAs whose activity depend on both RNA secondary structure and other higher ordered RNA interactions (Cochrane and Strobel 2008). There are many different classes of ribozyme (Rbz), each exhibiting a different catalytic structure, with hammerhead Rbzs and the hepatitis delta Rbz arguably being both the most intensively used for research purposes and studied (Peng et al. 2021). We reasoned that embedding a Rbz in the [−] RNA and monitoring the extent to which this strand is subsequently cleaved would provide a direct in situ readout of RNA folding. In this study we used Rbzs to assess RNA folding within the [−] RNA of hepatitis C virus (HCV), feline calicivirus (FCV), chikungunya virus (CHIKV), hepatitis E virus (HEV), human rhinovirus (HRV) and yellow fever virus (YFV). Our results confirm that the [−] strands of some positive strand RNA viruses are able to form functional RNA structures. Excitingly, we also demonstrate that this is not conserved across all positive strand RNA viruses but is specific to different divergent families and genera. Finally, our data suggest that for sequences in the [−] RNA to adopt a structural fold, active participation of host and/or viral proteins is required.

## RESULTS

### Cleavage of an HCV replication intermediate by a *cis*-acting hepatitis delta virus (HdV) Rbz

**FIGURE 1.**
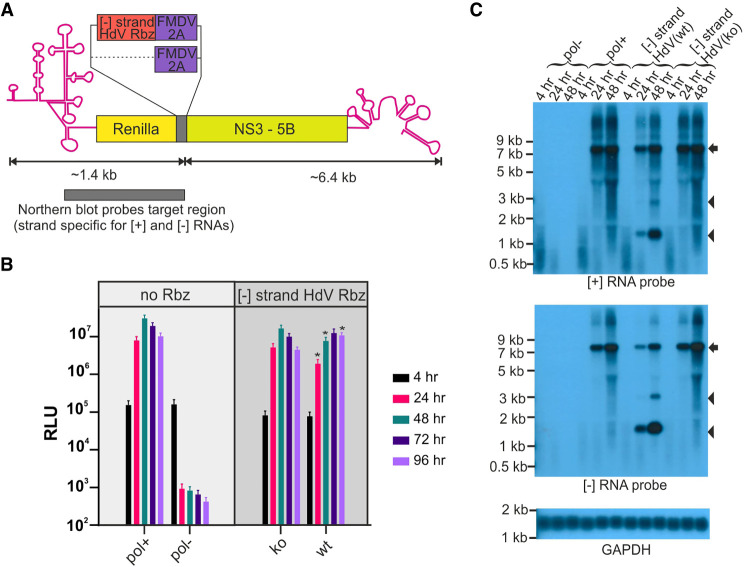
Placing the HdV Rbz in the [−] RNA of HCV cleaves this RNA and suppresses replication. (*A*) Schematic depiction of the HCV gt2a replicon encoding the HdV Rbz in its [−] RNA. Image includes the region of both [−] and [+] strands recognized by the strand-specific probes used for northern blotting. (*B*) Replication of constructs carrying either an active (wt) or inactive (ko) HdV Rbz in their [−] RNA. Included are replication-competent and replication-defective controls lacking the inserted Rbz sequence. Significant differences between HdV(ko) and (wt) constructs are highlighted ([*] *P* < 0.05; paired *t*-test; *n* = 6). (*C*) Northern blot of RNA from transfected cells. The arrow highlights the position of full length transcripts and the arrow heads the position of products produced as a result of Rbz activity.

To assess the potential for Rbzs to fold and cleave the [−] RNA of a positive strand RNA virus in *cis*, we initially chose to focus on HCV. The basis for this decision was because of evidence that HCV allows folding of relatively complex native structures in its [−] RNA (Friebe and Bartenschlager 2009; Schult et al. 2019). Additionally, we opted to use subgenomic replicon-based constructs because they enable the relevant stages of virus genome replication to be studied in isolation from other stages of the virus replicative cycle—such as entry, packaging and egress. A monocistronic HCV genotype 2a (gt2a) replicon (JFH1DVR-mono) was selected that expressed both a Renilla-FMDV2A luciferase reporter fusion protein and the HCV viral replicase (NS3-5B) needed for RNA replication. It was subsequently modified to introduce a reverse complemented HdV Rbz sequence (84 nt) between the Renilla and FMDV2A coding regions, generating HCVgt2a_HdV(wt) (Fig. 1A). A mutated HdV Rbz, with nucleotide substitutions designed to disrupt the folding of this RNA and hence its activity, was introduced into the [−] strand of an otherwise identical replicon to generate a second control construct, HCVgt2a_HdV(ko). Both constructs had the reverse complemented HdV sequence positioned within their coding region in such a way that the ORF was maintained, and the foreign peptide encoded by the insertions was identical. Cells were transfected with these two constructs as well as the original JFH1DVR-mono construct and a polymerase-defective control construct, and luciferase activity was measured over time as a readout of replication activity (Fig. 1B).

Four hours post-transfection (hpt), when the luciferase activity arises from translation of the input RNAs alone, the signal was twofold lower for both the HdV constructs compared to JFH1DVR-mono and replication-defective control constructs. Given that no replication would have occurred at this point, as illustrated by the signal being the same between the replication-competent and -defective controls, this difference likely comes from partial disruption of luciferase activity arising from the insertion of the reverse complemented HdV Rbz sequence into the Renilla-FMDV2A fusion protein. After this 4 h time point, HCVgt2a_HdV(wt), HCVgt2a_HdV(ko), and JFH1DVR-mono all produced an initial increase in luciferase over time compared to a decrease seen for the polymerase-defective control construct, demonstrating that all three former RNAs were replication-competent. However, the luciferase signal from the replicon with the active Rbz significantly diverged from its inactive Rbz counterpart as the latter produced a more rapid rise in luciferase activity, with luciferase levels peaking at 48 versus 96 hpt. This coincided with the observation that replication of HCVgt2a_HdV(wt) was suppressed, with luciferase levels at 32% and 44% of those produced by its HdV(ko) counterpart at 24 and 48 hpt. While at 96 hpt this trend was reversed, the likely explanation is that this was due to HCVgt2a_HdV(ko) transfected cells exhibiting earlier cytopathic effects and luciferase levels in this experimental group starting to drop.

If Rbz activity was suppressing replication, then cleavage of the [−] RNA would be anticipated. To establish whether this was the case, RNA was recovered from these same experiments and assessed by northern blot (Fig. 1C). Strand-specific probes directed at the Renilla coding region were unable to detect the input RNA at 4 hpt. However, full length [+] and [−] RNA could be detected at 24 and 48 hpt for all replication-competent constructs but not the replication-defective control. Consistent with the luciferase replication data, the abundance of these two RNAs were somewhat reduced in the HCVgt2a_HdV(wt) compared to HCVgt2a_HdV(ko) and JFH1DVR-mono transfected cells. The appearance of detectable levels of replicon transcript in HCVgt2a_HdV(wt) transfected cells at 24 h onward also coincided with the appearance of two other smaller RNA products that were absent from all other experimental groups. Importantly, the size of the most prominent of these matched the size of the [−] RNA fragment that was expected should Rbz cleavage be occurring. Interestingly, a similar size band also appeared in the genomic blot, indicating that the cleaved [−] RNA might serve as a template for RdRp activity. The identity of the second minor RNA species is not clear and interestingly it was not always observed. However, given that it was approximately twice the size of the cleaved 3′ end of the [−] RNA, and was picked up by both probes, it may represent a copy-back product.

### Improved cleavage of the [−] RNA strand using hammerhead Rbzs

**FIGURE 2.**
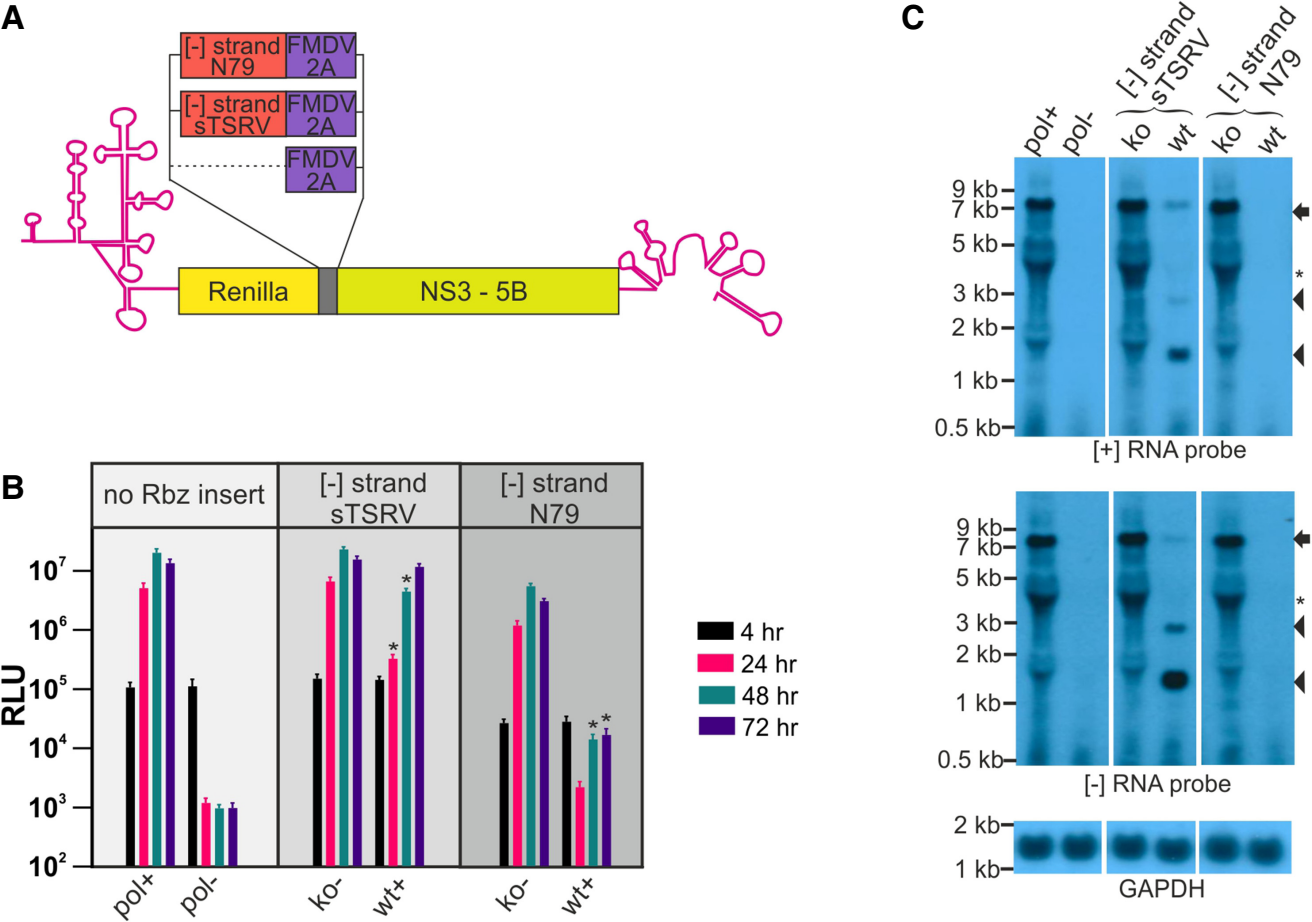
Hammerhead Rbzs are highly effective at cleaving the [−] RNA of HCV replicon constructs. (*A*) Schematic depiction of the HCV gt2a replicons used in this experiment. (*B*) Replication of constructs carrying either an active (wt) or inactive (ko) sTRSV or N79 hammerhead Rbz in their [−] RNA. Included are replication-competent and replication-defective controls lacking the inserted Rbz sequence. Significant differences between sTRSV(ko) and (wt) constructs, and between N79(ko) and (wt) constructs, are highlighted ([*] *P* < 0.05; paired *t*-test; *n* = 3). (*C*) Northern blot of RNA from cells transfected with replicon constructs 48 h earlier. The arrow highlights the position of full length transcripts and the arrowheads the position of products produced as a result of Rbz activity. Other bands on the gel (*) coinicident with the position of ribosomal RNAs, represent background artifacts.

Having used the HdV Rbz to show that functional structure was able to form in the HCV [−] RNA, we were interested in whether other smaller Rbzs with complex secondary structure could be used to further enhance cleavage efficiency. The monocistronic HCV gt2a replicon, JFH1DVR-mono, was again adapted so as to contain one of two different reverse complemented hammerhead Rbzs placed between the Renilla and FMDV 2A coding regions. As before, the ORF of the replicon was maintained and control constructs encoding inactive versions of the same Rbz sequence in their [−] RNA were generated by introduction of synonymous mutations. The two Rbz sequences selected for analysis were from satellite RNA Tobacco Ringspot Virus (sTRSV; 49 nt) (Khvorova et al. 2003) and another derived from *S. mansoni* that had been further engineered for enhanced cleavage activity (N79; 83 nt) (Yen et al. 2004). The four constructs generated were HCVgt2a_sTRSV(wt), HCVgt2a_sTRSV(ko), HCVgt2a_N79(wt), and HCVgt2a_N79(ko). Based on luciferase measurements, replication of constructs carrying the active versions of these Rbzs was significantly suppressed (Fig. 2A,B), more so than constructs bearing the HdV Rbz. In contrast, the inactive Rbz control constructs demonstrated robust replication, which after normalizing to the input signal at 4 h matched that of JFH1DVR-mono (data not shown). Renilla values produced from HCVgt2a_sTRSV(wt) were 5%, 20%, and 78% of those produced from HCVgt2a_sTRSV(ko) at 24, 48, and 72 h. More marked was the suppression seen with the N79(wt) Rbz where luciferase activity was found to be 0.1%, 0.2%, and 0.4% of the N79(ko) control levels at 24, 48, and 72 h, respectively. Northern blot analyses of RNAs taken from cells 48 h post-transfection and hybridized to probes complementary to the Renilla encoding region of the [+] and [−] strands identified sTRSV Rbz cleaved products (Fig. 2C). The relative abundance of these products compared to their full length uncleaved [+] and [−] RNA counterparts was notably increased compared to that seen with the HdV Rbz, confirming that the sTRSV Rbz was indeed more efficient at cleaving the [−] strand of HCV. Also consistent with the luciferase data, the overall levels of transcript were much lower in cells transfected with HCVgt2a_sTRSV(wt) versus HCVgt2a_sTRSV(ko). No replicon RNA signal was detected in cells transfected with HCVgt2a_N79(wt). Given that replication of HCVgt2a_N79(ko) was robust, this is presumably because of efficient N79 Rbz cleavage suppressing replication levels to below that detectable by northern blot.

### Rbz mediated suppression of HCV replication is positionally independent

**FIGURE 3.**
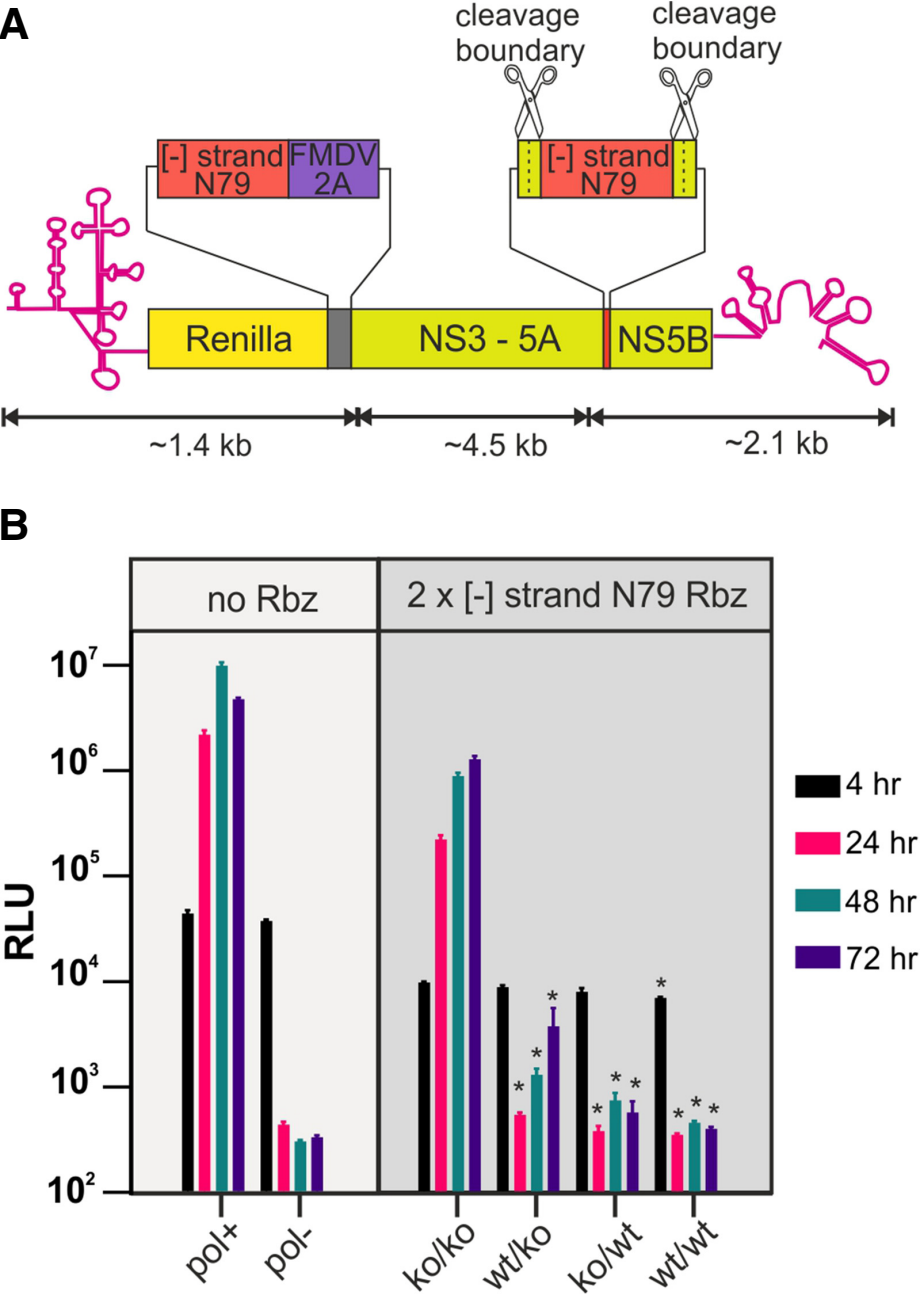
Rbzs embedded in the [−] RNA of HCV suppress replication irrespective of the position they are located at. (*A*) Schematic depiction of HCV gt2a replicons carrying two copies of the N79 Rbz positioned at two different locations within their [−] RNA. (*B*) Replication data from the constructs depicted in *A* as well as from replication-competent and replication-defective controls lacking the inserted Rbz sequence. Significant differences between the (ko/ko) construct and other 2× N79 containing constructs are highlighted ([*] *P* < 0.05; one-way ANOVA; *n* = 3). No significant differences were observed between other 2× N79 experimental groups.

In order to confirm the availability of the [−] RNA strand to form functional RNA structure in other regions of the virus genome during HCV replication, the positional independence of the Rbz was investigated. To achieve this, a second reverse complemented active N79 or inactive N79 sequence was introduced into the NS5A/5B boundary of both HCVgt2a_N79(wt) and HCVgt2a_N79(ko) (Fig. 3A). This was done in such a way that the NS3-5B replicase would still be expressed and the peptide resulting from this insertion would be cleaved away from both NS5A and NS5B. The resulting four constructs thus carried two separate copies of the N79 reverse completed ribozyme sequence, such that none [HCVgt2a_2xN79(ko/ko)] one [HCVgt2a_2xN79(wt/ko) and HCVgt2a_2xN79(ko/wt)], or both [HCVgt2a_2xN79(wt/wt)] were functional. All four RNAs along with controls were transfected into cells and luciferase activity monitored. Consistent with the N79 Rbz being active at both sites, transfection with either HCVgt2a_2xN79(wt/ko) or HCVgt2a_2xN79(ko/wt) resulted in a profound drop in luciferase activity over time, with this being more pronounced in the latter construct such that luciferase values were comparable to those of the replication-defective control (Fig. 3B). HCVgt2a_2xN79(wt/wt) also produced comparable luciferase values to the negative control whereas HCVgt2a_2xN79(ko/ko) showed increasing luciferase activity for the first 48 h of the assay that was significantly raised above that of the other 2xN79 constructs. Normalizing the signals to the 4 h time points suggested that HCVgt2a_2xN79(ko/ko) had slightly reduced replication capacity to that of JFH1DVR-mono, likely resulting from the inserted sequence placed between the duplicated NS5A/5B boundary (data not shown). However, given it was still clearly replication-competent we conclude that the lack or near lack of replication seen with HCVgt2a_2xN79(wt/ko), HCVgt2a_2xN79(ko/wt), and HCVgt2a_2xN79(wt/wt) is a direct consequence of N79 Rbz activity.

### Assessing the impact of the N79 Rbz on the replication of other positive strand RNA virus constructs

**FIGURE 4.**
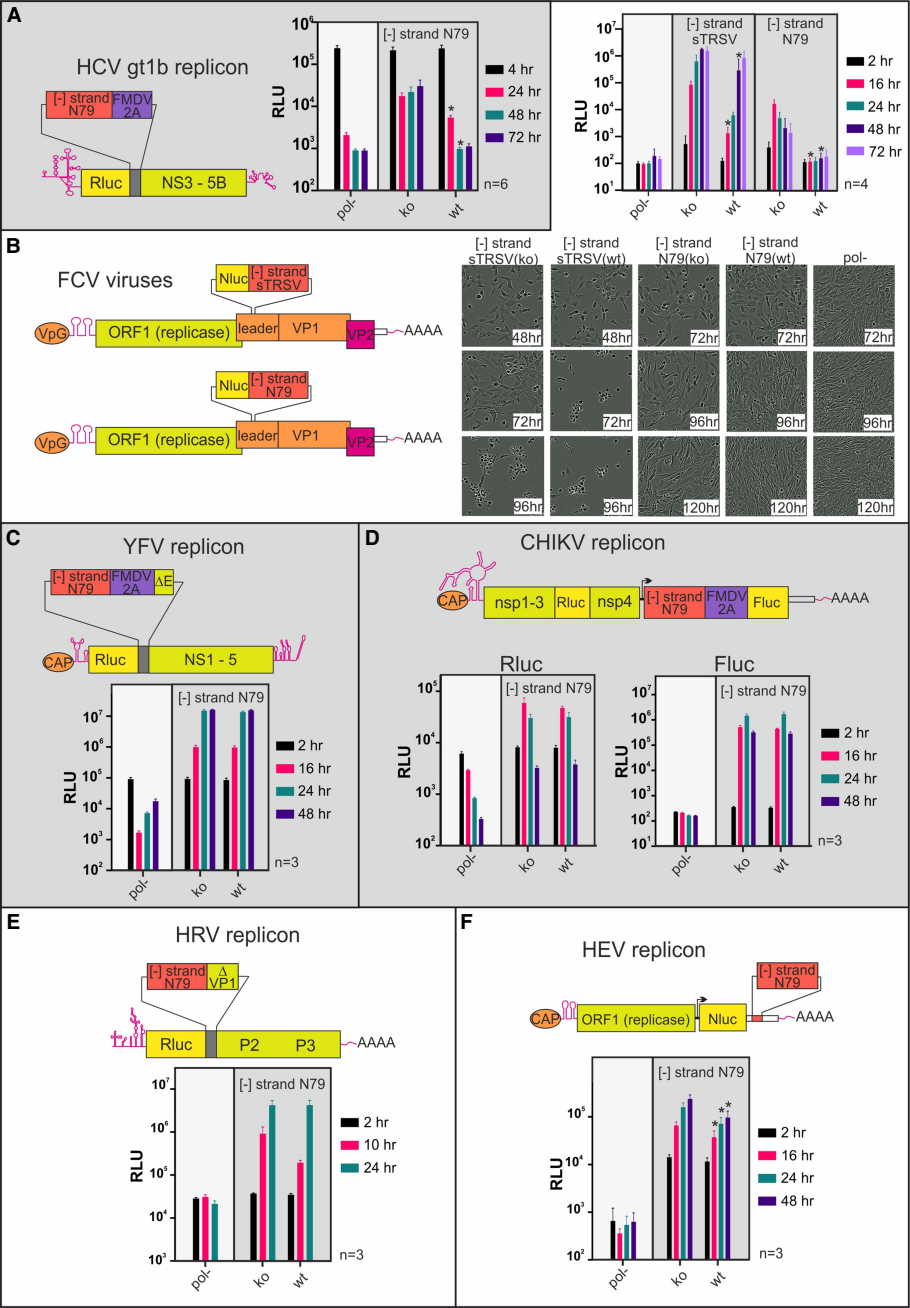
Embedding Rbzs in the [−] RNA of a diverse set of positive strand RNA virus constructs results in different replicative outcomes. A schematic depiction showing the positioning of the reverse complemented N79 Rbz or sTRSV Rbz sequence is provided for an (*A*) HCV gt1b replicon, (*B*) FCV virus, (*C*) YFV replicon, (*D*) CHIKV replicon, (*E*) HRV replicon, and (*F*) HEV replicon. The regions ΔE and ΔVP1 in the YFV and HRV schematics represent the carboxy-terminal ends of the E protein and VP1 protein, needed for proteolytic processing at the amino terminus of the NS1 and P2 boundaries, respectively. Also shown are luciferase replication data from those same isolates, and in the case of the FCV virus experiments representative images illustrating differences in cpe development over time. The number of experimental repeats for each luciferase assay is indicated next to the respective graph. Significant differences between inactive (ko) and active (wt) Rbz constructs are highlighted ([*] *P* < 0.05; paired *t*-test).

Having established that the N79 Rbz effectively suppressed replication of the HCV gt2a replicon, we assessed its impact on replication when placed in the [−] RNA of other positive strand RNA virus constructs. A variety of HCV genotype replicons exist although they display reduced levels of replication compared to JFH-1 gt2a constructs. Introduction of a functional N79 reverse complemented sequence into a HCV gt1b replicon abolished replication whereas an inactive N79 Rbz did not (Fig. 4A).

We next examined Rbz activity in the [−] RNA of a member of the *Caliciviridae* family; viruses which harbor relatively large stem–loops in this strand that function as a subgenomic promoter (Simmonds et al. 2008). Because *Caliciviridae* replicons have limited replicative capacity, reverse complemented sTRSV and N79 sequences were instead introduced into the leader of the capsid protein sequence of an infectious clone of FCV, a location previously shown to tolerate foreign sequence (Abente et al. 2010). A NanoLuc coding region was also introduced at this location to simplify monitoring of replication. Transfection of these constructs into cells produced different results depending on the Rbz sequence used (Fig. 4B). For constructs carrying either an active or inactive sTRSV Rbz, transfected cells exhibited cytopathic effects (cpe), suggestive of infectious virus production. Importantly, for sTRSV(ko) transfected cells the appearance of cpe always occurred sooner than in sTRSV(wt) transfected cells, although the exact timings of when it was seen did vary between experiments. This earlier appearance in cpe seen for the sTRSV(ko) construct paralleled what was observed for the luciferase activities, with this same construct producing significantly higher levels than sTRSV(wt) at all time points up to and including 48 h. In contrast, FCV constructs carrying the N79 Rbz sequences in their [−] RNA failed to produce cpe, although there was a suggestion that cell growth was perhaps suppressed, more so for the N79(ko) construct. Monitoring luciferase activity demonstrated that genome replication was occurring, at least for the N79(ko) construct, as luciferase levels increased over the first 16 h to ∼100-fold above background levels and then slowly decreased. In contrast, luciferase activity of the N79(wt) and the replication control construct were similar, demonstrating that the active N79 Rbz predominately blocked replication; a result comparable to what had been observed in the HCV constructs.

Having established that the reverse complemented N79 Rbz effectively blocked replication of HCV and FCV, we introduced it into other positive strand virus replicons in order to investigate the conservation of [−] RNAs ability to form functional RNA structures across divergent viruses. Interestingly, for a replicon derived from the 17D vaccine strain of YFV, no difference in replication could be discerned between constructs carrying the functional versus nonfunctional N79 sequence (Fig. 4C). A similar observation was made with the CHIKV replicon, where the Firefly luciferase signal (indicative of subgenomic RNA production) and the Renilla signal (indicative of genomic RNA transcription) were the same for both the functional and inactive N79 constructs at all time points tested (Fig. 4D). The situation was slightly different for HRV, where introduction of a functional N79 Rbz caused a consistent drop (55%–88%) in luciferase activity at 12 h in cells transfected compared to an inactive N79 control construct (Fig. 4E). However, this difference between the two constructs did not reach significance and disappeared at the 24 h time point when luciferase activities had peaked. In contrast, replication of the HEV replicon carrying the functional N79 Rbz was significantly impaired, generating a luciferase signal at 24, 48, 72, and 96 h post-transfection that was 92%, 60%, 53%, and 44% of the signal produced from the inactive N79 control (Fig. 4F). Notably, these experiments showed that irrespective of whether an active N79 Rbz suppressed replication in these four viruses, the reduction in replication, if any, was slight in comparison to either FCV or HCV where N79 imposed a complete or near-complete block on replication.

**FIGURE 5.**
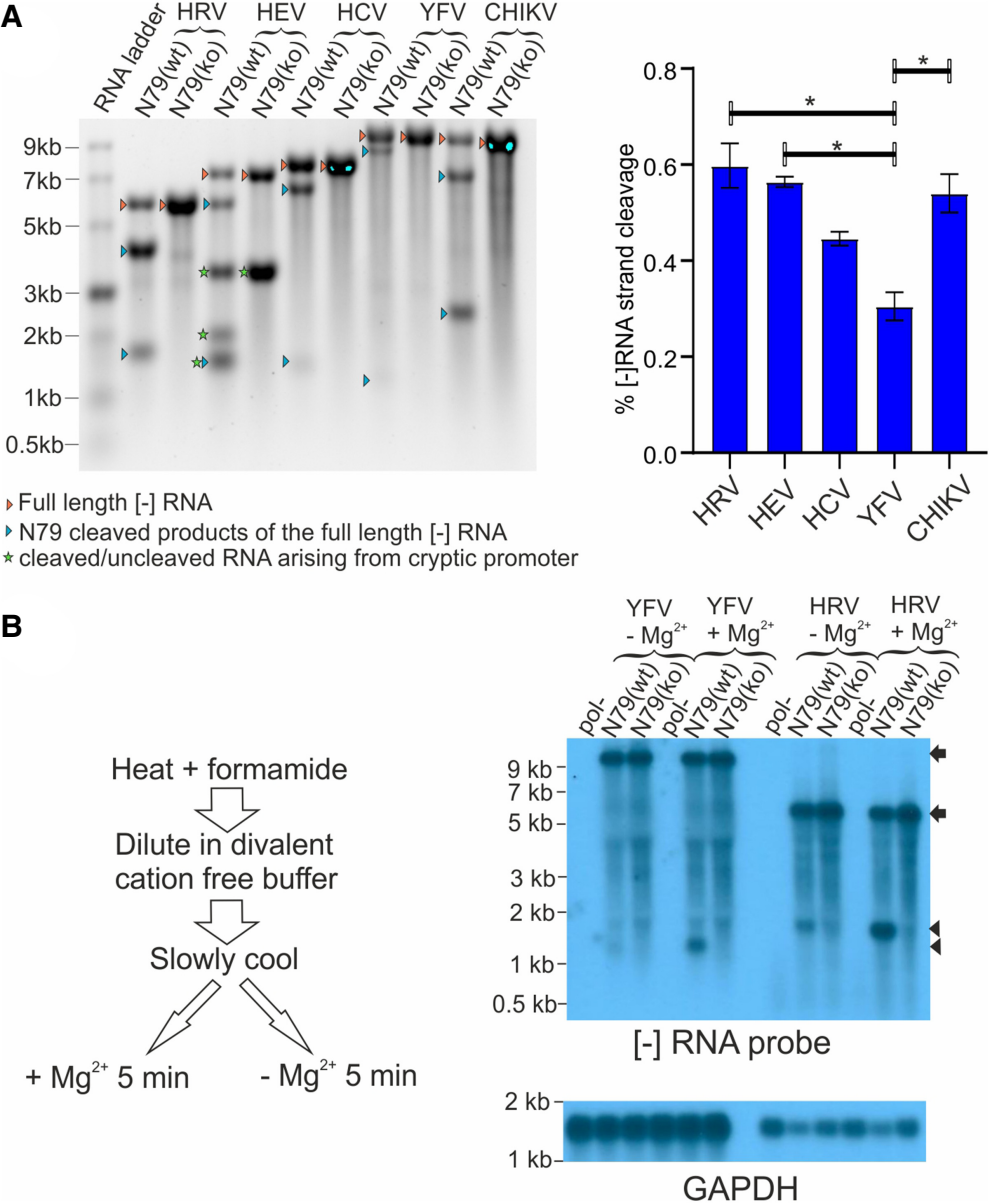
The N79 Rbz is not restricted in its ability to cleave the [−] RNA of positive strand RNA virus constructs when this RNA is in a single-stranded state. (*A*) Single-stranded [−] RNAs from replicons containing a reverse complemented N79 sequence were generated by in vitro transcription and their cleavage was assessed by gel electrophoresis and image capture. Significant differences between experimental groups are highlighted ([*] *P* < 0.05; one-way ANOVA; *n* = 3). (*B*) RNAs from cells transfected 24 h earlier with YFV or HRV replicons carrying a reverse complemented N79 sequence were collected. They were subsequently subject to treatment designed to release the [−] RNA from its double-stranded state and enable it to fold before Mg^2+^ was added to activate Rbz activity. Experimental groups lacking Mg^2+^ addition were included as controls. Cleavage was assessed by northern blot analysis . The arrows represent the position of full length transcripts and arrowheads the position of N79-cleaved product.

Rbz folding and hence activity can be influenced by adjacent sequences. Care had been taken to minimize the chance of surrounding sequences interfering with N79 activity by placing “insulator” sequences either side of the Rbz boundaries. However, it remained possible that mis-folding could account for some of the differences observed between the viral constructs regarding N79 activity. To investigate this, we first looked at folding using in silico free energy minimization prediction using the UNAFold software version 3.4 (Markham and Zuker 2008). In all cases N79 folding was robust, with the critical single-stranded loops and regions that form the ribozyme pseudoknot being well predicted albeit in the case of the HRV replicon where the extended stem I region containing the additional loop was missing (see Supplemental Fig. S2). We also introduced an RNA polymerase promoter immediately downstream from the 3′ end of the genome in the plasmids encoding five of the N79-containing virus replicons to allow in vitro production of full length (gt2a HCV, HRV, HEV, CHIKV) or near full length (YFV) [−] RNA. Analysis of these RNAs showed that they experienced no more than a twofold difference in rates of cleavage, with cleavage ranging from 30% to 60% (Fig. 5A). Equally importantly, these relatively small differences did not correlate with how effectively the N79 Rbz suppressed replication in cellulo, given that the [−] strand of HCV was the second least efficiently cleaved RNA species.

Another scenario that might have accounted for poor cleavage is mutation of the Rbz sequence such that activity is rapidly lost. To exclude this possibility, cells were transfected with N79-containing HRV and YFV replicons and cellular RNA was recovered 48 h later. Samples were heat denatured to melt all dsRNA, then diluted and cooled to promote RNA folding. Half of each sample was supplemented with 1 mM Mg^2+^ to enable Rbz catalysis while the other half was not. Subsequent northern blot analysis using a probe complementary to the [−] RNA was used to assess cleavage of this RNA (Fig. 5B). In the absence of Mg^2+^ supplementation, both YFV N79(wt) and N79(ko) transcripts appeared uncleaved and were equally abundant. The relative abundance of full length HRV [−] RNA N79(wt) and N79(ko) transcripts was also broadly similar, although unlike YFV a low level of cleavage of the N79(wt) transcript was also observed, consistent with the transient inhibition of replication observed at early time points in the replication assay. Importantly, the addition of Mg^2+^ resulted in an increase in cleavage of the HRV N79(wt) [−] transcript and cleavage of a proportion of the YFV N79(wt) [−] transcript not seen for the equivalent RNAs carrying the N79(ko) Rbz. We conclude a functional Rbz sequence is maintained in the [−] RNA strand of HRV and YFV during the time that genome amplification is occurring, but its activity is suppressed by dsRNA formation.

### Duplex formation blocks Rbz-mediated cleavage of NS5B synthesized products in vitro

**FIGURE 6.**
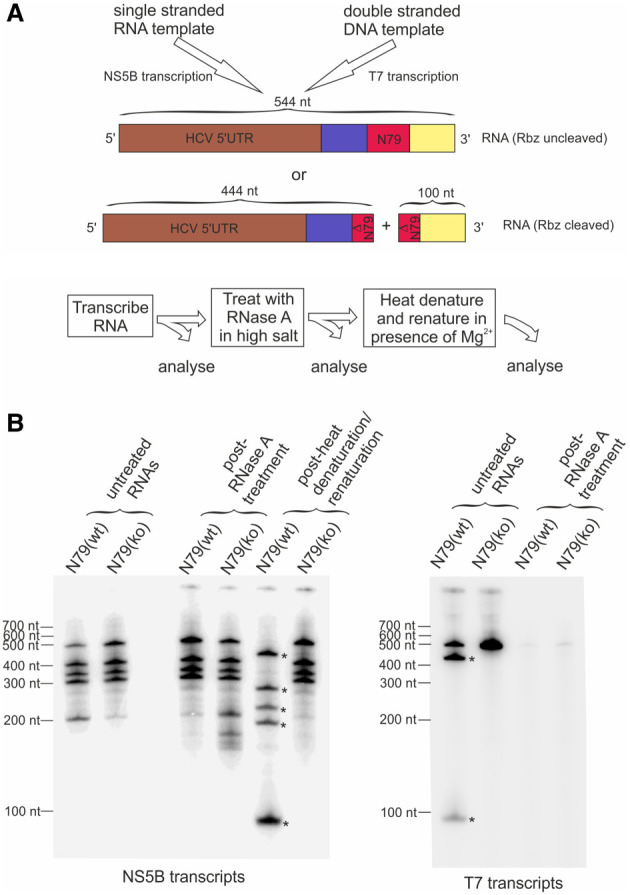
Rbz-containing RNAs are not cleaved when synthesized in vitro by the HCV RNA polymerase NS5B due to extensive base-pairing with the template strand. (*A*) Schematic depicting how NS5B and T7 polymerase transcription reactions were used to produce an N79 Rbz containing RNA. Templates were such that both sets of reactions were expected to transcribe identical RNAs, either encoding an active N79 Rbz sequence or one with a single nucleotide substitution in the active site [N79(wt) and N79(ko) respectively]. The expected sizes of RNAs produced, whether cleaved by Rbz activity or not, are shown. (*B*) All four [α-^32^P] labeled RNAs produced from the transcription reactions outlined in *A* were subjected to a series of treatments as detailed in the provided diagram before being run on a denaturing polyacrylamide gel. The high-salt RNaseA treatment step was used to selectively degrade single-stranded RNAs. The heat denaturation/renaturation step was used to melt dsRNA and allow single-stranded RNA folding. The asterisks indicate RNAs produced as a result of Rbz cleavage. Additional smaller bands in the NS5B transcription reactions likely arise from internal initiation. Results shown are representative of one of two experiments where initial transcription reactions were carried out at room temperature.

The extent that Rbz induced cleavage of the [−] RNA of HCV was much greater than most other constructs tested. To investigate whether RNA synthesized by the HCV viral polymerase in vitro was equally prone to Rbz cleavage and thus able to sample secondary structure, we performed a series of RNA polymerase reactions. HCV NS5B RNA-dependent RNA polymerase reactions were set up to produce a 544 nt RNA transcript containing either an active (wt) or inactive N79 (ko) Rbz (Fig. 6A). Parallel control T7 DNA-dependent RNA polymerase reactions were set up to produce these same two transcripts as single-stranded RNA molecules. Products were transcribed at both room temperature and 37°C. Analysis by denaturing polyacrylamide gel electrophoresis revealed that the full-length transcript produced by T7 had an apparent molecular weight that was ∼ 500 nt (Fig. 6B; Supplemental Fig. S3). Importantly, about half of the N79(wt) RNA produced by T7 was cleaved into products of a size consistent with that expected from N79 activity (444 and 100 nt). Indeed, this was specifically due to Rbz activity as the N79(ko) transcript produced by T7 remained uncleaved. In contrast, no obvious difference was observed between RNA products generated in the NS5B reactions when comparing between N79(wt) and N79(ko) experimental groups irrespective of the temperature of the transcription reaction; the RNAs present consisting of an approximately unit length (∼500 nt) product identical in size to the uncleaved RNA produced in the T7 reactions, as well as several smaller RNAs mostly ranging in size between 300–400 nt and likely to represent internal initiation products. To assess the double-stranded nature of the various polymerase reactions, all products were incubated with RNase A under high salt conditions. No degradation of the NS5B products was seen, indicating that they were duplexed with their template and therefore double-stranded. In contrast, the T7-derived products were highly sensitive to RNase A treatment. Finally, to confirm that dsRNA formation was preventing Rbz cleavage, the surviving RdRp product from the RNase treatment was subjected to heat denaturation and renaturation. In the N79(wt) experimental group, this resulted in a decrease in size of all RNAs by ∼100 nt as well as the appearance of an additional ∼90 nt band. In contrast, there was no change in appearance of the N79(ko) containing RNAs. Taken together these data show that in the absence of any other host or viral encoded activity, RNA produced by HCV NS5B is unable to even transiently access secondary structures that might be encoded within it.

## DISCUSSION

Knowledge of the different structural states that RNA can adopt in the replication complexes of positive strand RNA viruses remains incomplete. For members of the *Leviviridae* family which infect prokaryotes, and for members of *Narnoviridae* which infect fungi and insects, the [+] and [−] RNAs are kept in a predominately single-stranded state (Blumenthal and Carmichael 1979; Fujimura et al. 2005). In contrast, many other positive strand RNA viruses readily generate dsRNA when replicating (Weber et al. 2006; Son et al. 2015). Indeed, this is such a ubiquitous property that plants and animals have evolved detection systems for dsRNA, recognition by which stimulates an array of antiviral responses (Gantier and Williams 2007; Niehl and Heinlein 2019). However, whether the viral [−] RNA is in a predominately single-stranded or double-stranded state when contained within an active replication complex has only been effectively answered for a few select viruses because of technical challenges addressing this question. One issue is that infection produces dsRNA through processes other than those involved in productive genome replication (Richards et al. 1987). Both this and the presence of exhausted replication complexes have the potential to generate dsRNA decoys containing [−] RNA. Another difficulty is that active replication complexes are protected by lipid membranes. Typically, these need to be disrupted to gain experimental access to the RF and RI; a process which frustrates RNA structural analysis by promoting collapse of complementary RNAs into a double-stranded state (Richards et al. 1984). Nonetheless, for some viruses there is good evidence to suggest that the [−] RNA within RF and RI is in a double-stranded state (Panavas et al. 2006; Kovalev et al. 2014; Ertel et al. 2017; Klein et al. 2020). What is less clear is whether this is always the case. In this study, we sought to gain insight into this process by using Rbzs to assess RNA folding in the [−] RNA of various viruses. Our data show that the ability of a Rbz to cleave the [−] RNA of different viruses varies enormously. The simplest model explaining this observation is that the [−] RNA varies in the extent to which it is double-stranded in different viral RCs and it is this that correlates with both cleavage and the suppression of replication. Certainly, recent electron cryotomography studies on CHIKV replication spherules would support the notion that this virus [−] RNA is sequestered away in a double-stranded state, consistent with the lack of cleavage we observed in our studies (Laurent et al. 2022; Tan et al. 2022). Interestingly, these same studies suggest that the viral helicase nsp3 is either not associated with the replicase proteins found at the neck of the spherule, or if it is then this association in on the cytoplasmic rather than the lumen face. Thus the location of either host or viral helicases with respect to where the [−] RNA is sequestered could be behind much of the observations we have made. However, additional processes can be envisaged that could modulate cleavage independently of the double-stranded status the [−] RNA finds itself in. For instance, early cleavage of the [−] RNA during its synthesis, or while contained within the RF, could potentially have a more marked impact on replication compared to if it occurred once it was contained with the RI; dependent on the amount of new full length [+] RNA produced before cleavage of the [−] RNA within the RI took place. Both structural studies and our own in vitro transcription experiments (Fig. 6) suggest nascent RNA is bound to its template as it leaves the viral polymerase, and that this association is stable. However, we cannot exclude the possibility that coassociation of the viral polymerase with other viral and host proteins with strand separating properties could change this (Tuteja et al. 1995; Hirano et al. 2003; Kusakawa et al. 2007; Jennings et al. 2008). The physiological environment within the RC may also be different to that encountered in our in vitro experiments. Thus, transient sampling of encoded secondary structure may yet be possible for nascent [−] RNA as it exits the viral polymerase and if so our observations could reflect the extent to which this occurs. Under such circumstances, delaying passage of the viral polymerase through secondary structure present within the [+] RNA could then also modulate the extent to which cleavage occurs. Finally, it should be acknowledged that the lack of Rbz cleavage does not necessarily mean that a [−] RNA is in a double-stranded state, as constraints may still be placed on its folding when it is single-stranded. Indeed, the limited cleavage of the HRV [−] RNA by the N79 Rbz that we observed may reflect this, as the RI from poliovirus has previously been reported to be predominately single-stranded (Richards et al. 1984). Constraints on folding of the [−] RNA could arise from it being bound by viral proteins and explain why enterovirus such as poliovirus and HRV use structural elements at the 5′ of their [+] RNA as a promoter for [+] strand synthesis (Vogt and Andino 2010). Irrespective of the reasons, the fact that Rbz cleavage requires separation of [+] and [−] RNA means that where extensive cleavage is observed, the most likely explanation is that the [−] RNA either must be predominately single-stranded or be constantly fluctuating between a single- and double-stranded state.

The HCV RdRp, NS5B, produces dsRNA in vitro (Oh et al. 1999). Structural studies also suggest that newly synthesized products of NS5B leave the polymerase base-paired to their template (Appleby et al. 2015). Our results confirm that nascent NS5B products produced in vitro fail to sample secondary structure as a result of this base pairing. Consequently, folding within the [−] RNA of HCV most likely depends on additional viral or host encoded activities such as the HCV helicase, NS3. Helicase activity is achieved by the protein binding to a stretch of single-stranded RNA and migrating in a 3′–5′ direction, displacing any complementary RNA (Tai et al. 1996). Assuming NS3 is involved in opening up duplex structures in the RF and RI, one question that arises is how it accesses a region of single-stranded RNA to enable binding. Perhaps access is gained at the ends of these RNAs, possibly as a result of duplex breathing or mediated by terminal transferase activity of NS5B providing a single-stranded extension to one or other strands (Ranjith-Kumar et al. 2001). If so, NS3 helicase processivity within the RC must be considerable to allow sampling of RNA structure as far into the [−] strand as the position at which the Rbzs were placed. Another consideration is that once access is gained, any region of duplexed RNA opened up by the passage of NS3 still has the potential to reanneal once NS3 has moved on. As reannealing would reimpose a block on the [−] strand being able to fold, slowing or preventing it could also be important. It is possible that scaffolding activities provided by single-stranded RNA binding proteins such as NS4B and NS5A (Huang et al. 2005; Einav et al. 2008), as well as NS3 working as a functional oligomeric array (Sikora et al. 2008) play a role here. As RF and RI formation have yet to be formally demonstrated in HCV, it may even be possible that reannealing is prevented by physical separation of the [−] strand from its [+] counterpart. Indeed, in situ hybridization findings suggest that [−] RNA fails to colocalize with [+] RNA in HCV infected cells (Shulla and Randall 2015; Liu et al. 2019). However, it is difficult to reconcile such physical separation with the accepted model of HCV replication, that of an ER-derived double membrane vesicle harboring both [+] and [−] RNA strands (Paul et al. 2014). Whatever mechanisms HCV and other viruses use to enable sampling of complex structures within their [−] RNA, a better understanding of these could provide novel therapeutic angles for treatment.

Production of a subgenomic RNA resulting from internal initiation within a full length [−] RNA is a feature common to the *Caliciviridae* (Yunus et al. 2015), *Togaviridae* (Sawicki et al. 1978), and *Hepeviridae* virus families (Varma et al. 2011). In the case of the *Caliciviridae* family, genetic and biochemical evidence supports the involvement of a stem–loop found at the end of ORF1 as being critical in promoter function (Simmonds et al. 2008; Yunus et al. 2015). For the *Togaviridae* family and other viruses within the so-called alphavirus super family, the role that RNA structure plays in subgenomic promoter activity is disputed (Siegel et al. 1997; Adkins and Kao 1998; Haasnoot et al. 2000, 2002; Sivakumaran et al. 2004; Skov et al. 2012). Subgenomic RNA production by members of the *Hepeviridae* family has been less extensively studied, in part because of historically less tractable cell culture systems. Nonetheless, the region within the viral genome harboring the promoter has been identified (Ding et al. 2018), and [−] RNA conserved stem–loop structures can be found at this location (Cao et al. 2010). Given the lack of impact N79 had on CHIKV, our study lends weight to the hypothesis that genomic and subgenomic promoters within the [−] RNA of alphaviruses and *Togaviridae* more generally are recognized by their primary sequence. In contrast, the notable suppression of FCV by N79 and sTRSV Rbzs is consistent with the use of stem–loop structures in the [−] RNA of the *Calicivirdae* family serving as subgenomic promoters. Suppression of HEV replication by the N79 Rbz leaves open the possibility that structural elements harbored within the [−] RNA of *Hepeviridae* family members also serve as subgenomic promoters. However, it is notable that the extent to which HEV appears to sample RNA structure in this strand is far less than that of FCV.

Replication of West Nile Virus (WNV) has been suggested to depend on host cell proteins TIA-1 and TIAR binding to a 75 nt stem–loop found within the 3′ end of the [−] strand (Emara et al. 2008). The helicase expressed by this and other flaviviruses also belongs to the same DEAD-box helicase superfamily as that expressed by HCV (Byrd and Raney 2012). Therefore, it was notable that introduction of the N79 Rbz, a RNA of similar size to the stem–loop in the 3′ end of the [−] strand of YFV, had no impact on the replication of this virus. One of several reasons could account for this finding. Firstly, YFV and other flaviviruses may only be able to effectively sample RNA structure at the very ends of their [−] RNA because of reduced helicase processivity (Jennings et al. 2009; Xu et al. 2019). Alternatively, reliance on TIA-1 and TIAR binding may not be a universal feature of flaviviruses. Finally, it is possible that the predicted terminal stem–loop found at the end of the [−] RNA is not functional. Reverse genetics experiments looking at functional importance of putative stem–loop functions at the terminal ends of the [−] RNA of positive strand viruses is fraught with difficulties. These complications arise because of the risk of off-target effects on structures within the genomic strand as well as the need to avoid synonymous changes in coding regions. While the lack of Rbz activity in the [−] RNA strand cannot be used to disprove the existence of structural elements elsewhere in this same RNA, when Rbz cleavage does occurs it provides valuable supportive evidence that structural elements elsewhere are likely to form.

Our observations of Rbz activity within the [−] RNA of HCV is consistent with the virus relying on a large structured promoter at the 3′ end of this same RNA species. Even so it was noticeable how much more active Rbzs were in the HCV constructs compared to many of the other viral constructs. This begs the question, why does HCV expend what is likely to be a considerable amount of resources and energy driving duplex separation within its replication complex when it does not produce subgenomic RNAs? Indeed, it seems strange that any virus would evolve a large structured promoter within its [−] RNA unless there was already preexisting evolutionary pressure driving stand separation. Perhaps by keeping its [+] and [−] strands in a more separated state, HCV reduces the chances of it being detected by dsRNA antiviral sensors. While the replication complex membranes protect the RF and RI from antiviral sensors in the cytosol, turnover/destruction of these organelles does expose them to endosomal TLR3, something that HCV already seeks to minimize by promoting RC secretion (Grünvogel et al. 2018). By being single-stranded when introduced into the endosome, complementary RNAs could be degraded before they form a more RNase-resistant double-stranded state and therefore avoid TLR3 recognition. It is also possible that the protection afforded by the RC against dsRNA sensors is not absolute (Uchida et al. 2014). Under such circumstances, having [−] and [+] RNAs in a more single-stranded state would certainly be beneficial. Although HCV and other hepaciviruses are unusual among positive strand RNA viruses in their ability to establish chronic infections with active viral replication in their immunocompetent hosts, they are not alone. Indeed, FCV achieves this (Wardley 1976), as does a number of other positive strand RNA viruses including members of the norovirus family (Thackray et al. 2007), pegiviruses (Thomas et al. 1998; Tomlinson et al. 2020), and pestiviruses (Becher and Tautz 2011). In the future it would be interesting to determine the extent to which Rbzs are active in the [−] RNA of a broader range of these viruses, as well as examine whether changes to RNA structural sampling within this strand might impact on host dsRNA sensing.

## MATERIALS AND METHODS

### Cell culture

HeLa (Ohio), CRFK and BHK21 cells were maintained in DMEM (Invitrogen) supplemented with 50 units penicillin, 50 mg streptomycin and 10% (v/v) fetal calf serum at 37°C and 5% CO_2_. Huh7.5 cells were maintained under similar conditions but with an additional supplementation of nonessential amino acids made to the media.

### DNA constructs

The location where the reverse complemented ribozyme sequence was positioned in each viral genome was based on practical considerations predominately focused around choosing an insertion point that was not going to disrupt viral replicase protein function or key structural RNA elements in the genome. The JFH-1 based monocistronic replicons, JFH1DVR-mono and JFH1mono (GAA), have been described (Gomes et al. 2015) and served as HCV gt2a replication functional and polymerase-defective controls lacking Rbzs. To insert the positive strand HdV Rbz sequence into JFH1DVR-mono, PCRs using primer pairs 1 + 2 (see Supplemental Table S1 for primer and synthetic DNA sequences) with template pMNV* (Ward et al. 2007), and primer pairs 3 + 4 and 5 + 6 with template JFH1DVR-mono were used to generate three overlapping products that were then combined in a second round PCR reaction with primer pairs 3 and 6. This product and a comparable DNA containing an inactive HdV Rbz, introduced using primer pairs 7 + 8, were cloned into the JFH1DVR-mono containing plasmid via BglII and RsrII restriction sites to generate plasmid constructs encoding HCVgt2a_HdV(wt) and HCVgt2a_HdV(ko).

To exchange the HdV Rbz for the sTRSV Rbz (Khvorova et al. 2003) and the cleavage optimized *Shistosoma mansoni* N79 Rbz (Yen et al. 2004), PCRs were performed with primer pairs 9 + 10 and 11 + 12 using HCVgt2a_HdV(wt) as a template. The two DNA products generated were used as templates alongside Ultramer oligonucleotides TRSV or N79 (Integrated DNA Technologies) in a second round of PCR using primer pairs 9 + 12. Resultant products were cloned directly into the HCVgt2a_HdV(wt) plasmid via BglII and RsrII restriction sites to generate HCVgt2a_sTRSV(wt) and HCVgt2a_N79(wt) containing plasmids. The same products were also further modified using another round of two-step PCR mutagenesis using internal primer pairs 13 + 14 or 15 + 16 alongside 9 and 12, before again cloning into the HCVgt2a_HdV(wt) plasmid to generate HCVgt2a_sTRSV(ko) and HCVgt2a_N79(ko) containing plasmids.

To add a second copy of the N79 Rbz between the NS5A and NS5B coding region of the HCVgt2a replicon, three first round PCRs were set up using primer pairs 17 + 18, 19 + 20, and 21 + 22. The template used in each reaction was either HCVgt2a_N79(wt) or HCVgt2a_N79(ko). All three products were combined in a second round PCR using primer pairs 17 + 22 and the resultant DNA cloned into both the HCVgt2a_N79(wt) or HCVgt2a_N79(ko), containing plasmids via SacII and SalI restriction sites to generate plasmids containing HCVgt2a_2xN79(wt/wt), HCVgt2a_2xN79(ko/ko), HCVgt2a_2xN79(wt/ko) and HCVgt2a_2xN79(ko/wt).

Insertion of the N79 Rbz into other replicon constructs was achieved as follows. For the HRV replicons, PCRs were undertaken using primer pairs 23 + 24 in combination with either HCVgt2a_N79(wt) or HCVgt2a_N79(ko) as a template, as well as primer pairs 25 + 26 in combination with pR16.11 (accession number L24917.1; a gift from T. Tuthill) as a template. The resultant sets of DNAs were combined in a second round PCR with primers 23 + 26 and the resultant product cloned into an in-house pR16.11 derived replicon (sequence available on request) via AscI and ClaI restriction sites to generate plasmids containing HRV_N79(wt) and HRV_N79(ko). A control replication-defective version of HRV_N79(ko) was generated by cutting the associated vector with NsiI, polishing with Phusion polymerase (NEB) and religating. For the YFV replicons, PCRs were undertaken using primer pairs 27 + 28 and 29 + 30 using pACNR-FLYF-17D (Bredenbeek et al. 2003) (a gift from P. Bredenbeek) as a template, as well as primer pairs 31 + 32 using either HCVgt2a_N79(wt) or HCVgt2a_N79(ko) as a template. The resultant three sets of DNAs were combined in a subsequent PCR with primer pairs 27 + 30 and the resultant product cloned into pACNR-FLYF-17D via NotI and MluI restriction sites to generate plasmids encoding for the YFV_N79(wt) and YFV_N79(ko) replicons. A control replication-defective version of YFV_N79(ko) was generated by cutting the vector with ClaI, polishing with Phusion polymerase and religating. For the CHIKV replicons, PCR products was generated using primer pairs 33 and 34 in combination with either YFV_N79(wt) or YFV_N79(ko) as templates. The resultant product was cloned into pSP6_ ChikRepI-PRlucSG-FlucWT or pSP6_ChikRepI-PRlucSG-FlucGAA (Roberts et al. 2017) via ApaI + BlnI to produce plasmids containing CHIKV_N79(wt), CHIKV_N79(ko) and CHIKV_N79(ko)GAA replicons.

For the HEV replicons, the GFP coding region from pSK-E2-GFP (Emerson et al. 2004) (a gift from P. Farci) was first replaced by nanoluciferase to generate a nanoluciferase expressing replicon. To achieve this, PCR was used to amplify the nanoluciferase open reading frame (ORF) using primers 35 + 36. The upstream HEV sequence was amplified by PCR using 37 + 38. The resulting PCR fragment was combined in a second round PCR and cloned using Xhol and EcoRI to generate pSK-E2-nLuc. To insert the Rbz into pSK-E2-nLuc, PCRs were undertaken using primer pairs 39 + 40 using either HCVgt2a_N79(wt) or HCVgt2a_N79(ko) as a template. The resultant product was cloned into pSK-E2-nLuc via EcoRI restriction sites to generate plasmids containing the replicons HEV-nLucN79(wt) and HEV-nLucN79(ko). A control replication-defective version of pSK-E2-nLuc containing an inactivating mutation to the RNA-polymerase active site (GDD > GNN) was generated by PCR using primer 41 + 42 and 43 + 44 with pSK-E2-nLuc as template. The resultant PCR products were combined in a second round PCR using primers 41 + 44 and cloned using Xhol and EcoRI to generate a plasmid containing the replicon HEV-nLuc-GNN.

For the HCV genotype 1b replicon, first round PCR products were generated using primer pairs 45 + 46 with template HCVgt2a_N79(wt) or HCVgt2a_N79(ko) and using primer pairs 47 + 48 with template pFKI341PVIlucUbiNS3-3′dgET (Friebe et al. 2005) (a gift from R. Bartenschlager). Resultant DNAs were used as templates in a second round PCR using primer pairs 45 + 48 and the products produced cloned into pFKI341PVIlucUbiNS3-3′dgET via NotI and BssHI restriction sites to generate plasmids encoding HCVgt1b_N79(wt) and HCVgt1b_N79(ko) replicons.

For the FCV constructs, pQ14 (Sosnovtsev and Green 1995) (a gift from K. Green) was used as a template in a two-step PCR using primer pairs 49 + 50 and 51 + 52. The first round products were pooled and used as a template with the primer pairs 49 + 52 to generate a DNA that was cloned back into pQ14 via BstBI and SpeI restriction sites, introducing a multiple cloning site (MCS) into the leader of the capsid sequence of FCV. GBlocks (Integrated DNA Technologies) encoding a Nanoluc_antisense_sTRSV fusion product or Nanoluc_antisense_N79 fusion product were then cloned into this MCS via KpnI and PstI restriction sites generating plasmids containing the viruses FCV_sTRSV(wt) and FCV_N79(wt). The GBlocks were also used as templates for a two-step PCR using internal mutagenic primers 13 and 14 or 15 and 16 to generate products that were cloned into the MCS, generating FCV_sTRSV(ko) and pQ14_FCV_N79(ko) containing plasmids. A replication-defective FCV construct was generated by cutting pQ14 with XhoI, polishing with Phusion polymerase and religating.

To modify plasmids containing the N79-containing replicon constructs so that they could be used to transcribe the [−] strand, these DNAs were first linearized with the restriction site found downstream and adjacent to their 3′UTR (NotI, XbaI, BglII, BamHI, and XhoI for CHIKV, HCVgt2a, HEV, HRV, and YFV constructs, respectively). A pair of complementary oligos containing either a SP6 (HCV, HEV, HRV, and YFV) or T7 (CHIKV) promoter and with overhanging ends compatible with those of the digested plasmid DNA were ligated into these same DNAs. The presence and orientation of the inserted DNA was confirmed by PCR and sequencing. For the purpose of identification, the names of the constructs produced bear the parental constructs name from which they derive but contain an additional “as” (antisense) prefix [e.g., asHCVgt2a-N79(wt)].

To generate constructs containing a reverse complemented N79 sequence to be used for transcribing template RNAs for in vitro NS5B RNA-dependent RNA polymerase (RdRp) assays, a two-step PCR was performed. Primer pairs 53 + 54 and 55 + 56 generated amplified product from HCVgt2a_N79(wt) and HCVgt2a_N79(ko) templates. These two reaction products were then combined in a second PCR reaction with primer pairs 53 + 56 and the resultant DNA cloned into pCR-Blunt (Invitrogen) before being excised using XbaI + EcoRI and cloned into XbaI + EcoRI cut pSGRJFH1luc, generating pT7HCV(3′-)341asN79^wt^ and pT7HCV(3′-)341asN79^ko^. Constructs were linearized with MfeI and polished with Mung Bean nuclease prior to use in T7 transcription reactions that generated RNA template for subsequent NS5B RdRp reactions.

To generate control constructs for producing the same RNA species to be synthesized in the NS5B RdRp reactions, but via T7 polymerase, PCRs were performed using the primer pair 57 + 54 in combination with pSGRJFH1luc as template DNA, and the primer pair 55 + 58 in combination with either HCVgt2a_N79(wt) or HCVgt2a_N79(ko) as template DNAs. First round products were combined in a second PCR using primers 57 and 58. Products were first cloned into pCR-Blunt, then excised with XbaI and EcoRI and cloned into XbaI + EcoRI cut pSGRJFH1luc to produce pT7HCV(5′+)341N79^wt^ and pT7HCV(5′+)341N79^ko^.

### Production of RNA transcripts from DNA templates

All RNAs other than HEV, FCV and radiolabeled transcripts (see RdRp assay protocol) were generated as follows. Five micrograms of linearized plasmid template were pretreated with RNA*secure* (Thermo Fisher) and used in a 50 µL transcription reaction containing 5× transcription buffer (Thermo Fisher), 8 mM rNTPs, 1.25 units RiboLock RNase Inhibitor (Thermo Fisher), and 60 units of either T7 or SP6 RNA Polymerase (Thermo Fisher). After an overnight incubation at 30°C, 2 units RNA-free DNase (NEB) was added, and reactions left at 37°C for 30 min. RNA transcripts were precipitated by addition of LiCl and the resultant pellet washed in 70% ethanol before resuspension in RNase-free water. Capping of YFV and CHIKV replicon RNA transcripts was performed using the Vaccinia Capping System (NEB) according to manufacturer's recommendations. HEV and FCV transcripts were generated using the HiScribe T7 ARCA mRNA kit (NEB) using 1 µg of linearized plasmid template following manufacturer's instructions. The integrity of all RNAs was verified by running RNAs on a MOPS-formaldehyde agarose gel and visualizing product by SYBR Gold Staining (Thermo Fisher). When required, relative quantification of RNA species on the gels was achieved using a ChemiDoc XRS+ Imager (Bio-Rad).

### Cell electroporation and monitoring of replicon replication

Huh7.5 cells were used to monitor replication of HCV, HEV, and YFV replicons, BHK21 used to monitor replication of CHIKV replicons, HeLa (Ohio) used to monitor replication of HRV replicons and CRFK cells used to monitor replication of FCV. Once cells had reached confluence they were detached, washed 2× in ice-cold RNase-free phosphate buffered saline (PBS) and resuspended at 1 × 10^7^ cells/mL in ice-cold PBS. Four hundred microliters of the cell suspension was mixed with 2 µg replicon transcript, transferred to a 0.4 cm cuvette, electroporated at 270 V, 960 µF (BioRad GenePulse II) and resuspended in 6 mL growth medium. Cells were plated out in 12-well plates and maintained at 37°C (HCV, YFV, CHIKV, HEV) or at 33°C (HRV) until harvested by washing in PBS and lysis in Passive Lysis Buffer (Promega). Luciferase activity was determined using either the Renilla Luciferase Assay Kit (Biotium), Nano-Glo Luciferase Assay System (Promega) or Dual Luciferase Reporter Assay (Promega). Live cell imaging was performed with an IncuCyte Dual Colour Zoom FLR (Essen BioScience) within a 37°C humidified CO_2_ incubator scanning hourly up to 120 h post-transfection, collecting multiple images per well. The .mp4 files generated had individual stills extracted from them using VLC media player.

### Northern blot

To generate probes, the HCVgt2a_HdV(wt) vector was used as a template in an asymmetric PCR reaction (Tang et al. 2006) containing a pair of outer primers (primers 59 and 60) and either an inner forward (primer 61) or inner reverse primer (primer 62). Single-stranded products produced by the reaction were separated from other reaction products on an agarose gel and purified. Resultant DNAs as well as a 316 bp GAPDH PCR product amplified from p-Tri-GAPDH (Thermo Fisher) were biotinylated using PlatinumBrightBIO (Kreatech) reagent. Total cellular RNA was recovered using TriFAST reagent (Peqlabs) according to manufacturer's instructions, run on a 0.8% MOPS-formaldehyde agarose gel, the gel stained using SYBR Gold to confirm rRNA integrity and the RNAs subsequently transferred to SensiBlot Plus nylon membrane (Fermentas). After UV-crosslinking, membranes were preblocked by a 30 min incubation in ULTRAhyb (Thermo Fisher) at 42°C, before an overnight incubation in Ultrahyb containing a biotinylated probe. An unbound probe was removed by washing the membrane at 42°C in 2× SSPE + 0.1% SDS and subsequently in 0.1× SSPE + 0.1% SDS. A bound probe was detected using the BrightStar Northern Blot Detection Kit (Invitrogen). Because probes could not be stripped from membranes, parallel blots were probed with GAPDH as a control. Images were captured on film. The specificity of both strand-specific probes was confirmed by hybridization to control northern blot membranes containing either HCV [+] or [−] strand in vitro RNA transcripts (Supplemental Fig. S1).

### RdRp assay

Five hundred nanograms of HCV(3′-)341asN79^wt^ and HCV(3′-)341asN79^ko^ RNA transcripts were heated to 90°C for 2 min in a final volume of 16.2 µL before slowly cooling to room temperature and placing on ice. To these RNA templates the following was added; 2.5 µL 10× NS5B transcription buffer (200 mM Tris-HCl [pH 7.5], 50 mM MgCl_2_, 10 mM DTT, 250 mM KCl, 1 mM EDTA), 0.6 µL RNaseOUT (Thermo Fisher), 1 µL 10 mM rATP, 1 µL 10 mM rCTP, 1 µL 10 mM rGTP, 1 µL 0.1 mM rUTP, 1 µL [α-32P]rUTP (10 µCi; 3000 Ci/mmol PerkinElmer) and 0.7 µL JFH1 NS5BΔC21 or JFH1 NS5BΔC21(GAA) at 0.3 mg/mL (Simister et al. 2009). Parallel T7 reactions performed at the same time used 1.25 µg XbaI linearized pT7HCV(5′+)341N79^wt^ and pT7HCV(5′+)341N79^ko^ as templates in a 25 µL final reaction volume containing 2.5 µL 10× T7 transcription buffer (NEB), 0.6 µL RNaseOUT, 1 µL 10 mM rATP, 1 µL 10 mM rGTP, 1 µL 10 mM rCTP, 1 µL 1 mM rUTP, 0.5 µL [α-32P]rUTP and 1 µL T7 polymerase (NEB). Experiments were performed where transcription was allowed to occur for 2 h at room temperature or at 37°C. Afterwards the NS5B RdRp reactions were placed on ice while the T7 reactions were supplemented with 1 unit RNase-free DNase and incubated at 37°C for 20 min. All samples were centrifuged through MicroSpin G-25 columns (Amersham) preequilibrated with 10 mM Tris (pH 7.5) 1 mM EDTA (TE) buffer before further addition of TE buffer to a final volume of 100 µL, phenol/chloroform extraction and ethanol precipitation in the presence of 20 µg glycogen. Half of each RNA was kept back for subsequent analysis while the other half was subjected to digestion with RNase A in a 100 µL high salt buffer (40 µg/mL RNase A, 10 mM Tris [pH7.5], 350 mM NaCl, 5 mM EDTA) for 60 min at 30°C before addition of 4 µL 20 mg/mL proteinase K and 6.6 µL 10%SDS and incubating at 37°C for 30 min. Reaction products were then phenol/chloroform extracted, ethanol precipitated, resuspended in 8 µL H_2_O and again half the recovered material was kept back for subsequent analysis. The remaining 4 µL of samples were mixed with 4 µL of formamide and the RNAs heated to 85°C for 2 min before being rapidly diluted by the addition of 196 µL Rbz cleavage buffer preheated to 85°C. Samples were allowed to cool slowly to room temperature before being subjected to ethanol precipitation in the presence of 20 µg glycogen as a carrier. RNAs from both treated and untreated arms of the experiment were separated on a 5% denaturing acrylamide:bisacrylamide (19:1) gel alongside a RiboRuler Low Range RNA Ladder (Thermo Fisher) and the gel fixed, stained with Methylene Blue and dried onto Whatman 3 MM filter paper. Gel images were captured by exposure to both CL-Xposure X-ray film (Thermo Fisher) or BAS-MP IP phosphoimager plate (Fujifilm) and scanned at 50 µm resolution at 635 nm in a FujiFilm FLA-5100 fluorescent imager analyzer.

## DATA DEPOSITION

Supplemental Table S1 and Supplemental Figures S1–S3 are provided as Supplemental Files. Raw luciferase data as well as original .jpg, .tiff and .mp4 files produced during this study are available at https://doi.org/10.5281/zenodo.5873054.

## SUPPLEMENTAL MATERIAL

Supplemental material is available for this article.

## Supplementary Material

Supplemental Material

## Figures and Tables

**Morgan Herod:**
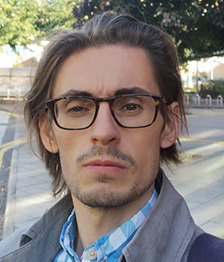


**Joseph Ward:**
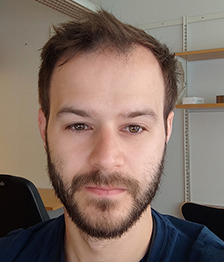


## MEET THE FIRST AUTHORS

***Meet the First Author(s)* is a new editorial feature within *RNA*, in which the first author(s) of research-based papers in each issue have the opportunity to introduce themselves and their work to readers of *RNA* and the RNA research community. Morgan Herod and Joseph Ward are the co-first authors of this paper, “Positive strand RNA viruses differ in the constraints they place on the folding of their negative strand.” Morgan is an MRC funded CDA fellow on transition to independence at the University of Leeds in the School of Molecular and Cellular Biology. His research focuses on the replication of positive-sense RNA viruses. Joe did this work as a post-doc at the University of Leeds and is now an Associate Principal Scientist at AstraZeneca in Gothenburg, Sweden.**

**What are the major results described in your paper and how do they impact this branch of the field?**

Single-strand positive-sense RNA viruses replicate their genome via a negative-sense RNA intermediate, the physical state of which is poorly understood when present in the infected cell. In this paper we used ribozymes to probe the nature of this intermediate. The major finding from our study is that not all families of positive-sense RNA viruses are able to access RNA secondary structures in their negative-sense RNA, implying that they use very different strategies for replication.

**What led you to study RNA or this aspect of RNA science?**

**MRH:** The negative-sense RNA intermediate produced by positive-strand RNA viruses is an essential part of their replication cycle. Understanding its nature, i.e., if it is free to form RNA secondary structures or has limitations imposed on it, has important implications for formation of viral replication organelles and the protein machinery they need to recruit.

**JW:** I think the principle of doing more with less is truly interesting. With minimal genome size and no DNA counterpart, RNA viruses have found clever ways of persisting. RNA structure is one of these examples which is fascinating to uncover how folding can produce function.

**During the course of these experiments, were there any surprising results or particular difficulties that altered your thinking and subsequent focus?**

When this work started, it was unclear whether these viruses would be able to sample foreign encoded structure within their negative-sense RNA, or if sampling would be restricted to only native RNA elements. The fact that we saw ribozyme-mediated cleavage in our early experiments with hepatitis C virus was exciting as there was a possibility that the approach would not work. The subsequent complete lack of ribozyme activity in yellow fever virus and chikungunya virus was also notable, indicating considerable diversity in replication strategies used.

**What are some of the landmark moments that provoked your interest in science or your development as a scientist?**

**MRH:** Having my first paper accepted was a real landmark moment for my career and gave me the confidence that I could conduct science to a publishable standard. My first post-doctoral position was also very important for my development as an experimental scientist.

**JW:** Having inspirational teachers and mentors at the early stages of my career and undergraduate days gave me the drive to dive into virology.

**If you were able to give one piece of advice to your younger self, what would that be?**

**MRH:** Working in collaborations or teams is much more insightful, powerful, productive, and enjoyable than working on your own.

**JW:** Sometimes more is not more! While it is good to have multiple interests and ideas, be careful not to let it drive focus away from progressing a project.

**Are there specific individuals or groups who have influenced your philosophy or approach to science?**

**MRH:** I feel fortunate enough to have had many excellent supervisors and mentors who have influenced my attitude and approach to science. To name one individual feels like not enough, but if I had to name one person, I think that Professor David Rowlands has probably had the largest influence on my scientific career and approach to science.

**JW:** When I started my PhD I was lucky enough to enter a multi-institute consortium. Working with such a breadth of researchers from different places provided me with a great education early on. I think it's also important to acknowledge the influence my undergraduate laboratory placement had on my career path; as such, Dr Jamie Kelly would need to be mentioned for inspiring me to travel this road.

**What are your subsequent near- or long-term career plans?**

**MRH:** My near-term career plans are to continue to establish and expand an independent research group. Longer term, I would like to apply for senior fellowships, which will allow the research group to establish leadership at an international level

**JW:** I have since stepped into the industrial field. My immediate plans are to continuing learning new skills and dive into new areas of RNA research to expand my knowledge as a scientist. Long term, I would like to develop collaborations between industry and academia to push and perform exciting science.

**What were the strongest aspects of your collaboration as co-first authors?**

We have quite overlapping but complementary sets of laboratory skills and experience, which means that we can work together as a team quite effectively. It doesn't rely on one person to do a certain set of tasks or experiments; we can each take the lead as work and time dictate.

**How did you decide to work together as co-first authors?**

We have worked alongside each other for a number of years so know we can communicate well and work reliably together. These skills are very important to us when working in partnership or as part of a team.
