# Supplementary material for: Positive strand RNA viruses differ in the constraints they place on the folding of their negative strand
Source: RNA. 2022 Oct;28(10):1359–76. doi: 10.1261/rna.079125.122 (PMC9479745; doi:10.1261/rna.079125.122)
Supplement: Supplemental Material [file supp_28_10_1359__DC1.html]

Positive strand RNA viruses differ in the constraints they place on the folding of their negative strand — Supplemental Material 

# Positive strand RNA viruses differ in the constraints they place on the folding of their negative strand

## Supplemental Material

- Supplemental\_Fig\_S2.pdf
- Supplemental\_Fig\_S3.pdf
- Supplemental\_Table\_S1.xlsx
- Supplemental\_Fig\_S1.pdf
