# Supplementary material for: Positive strand RNA viruses differ in the constraints they place on the folding of their negative strand
Source: RNA. 2022 Oct;28(10):1359–76. doi: 10.1261/rna.079125.122 (PMC9479745; doi:10.1261/rna.079125.122)
Supplement: Supplemental Material [file supp_079125.122_Supplemental_Fig_S2.pdf]

A

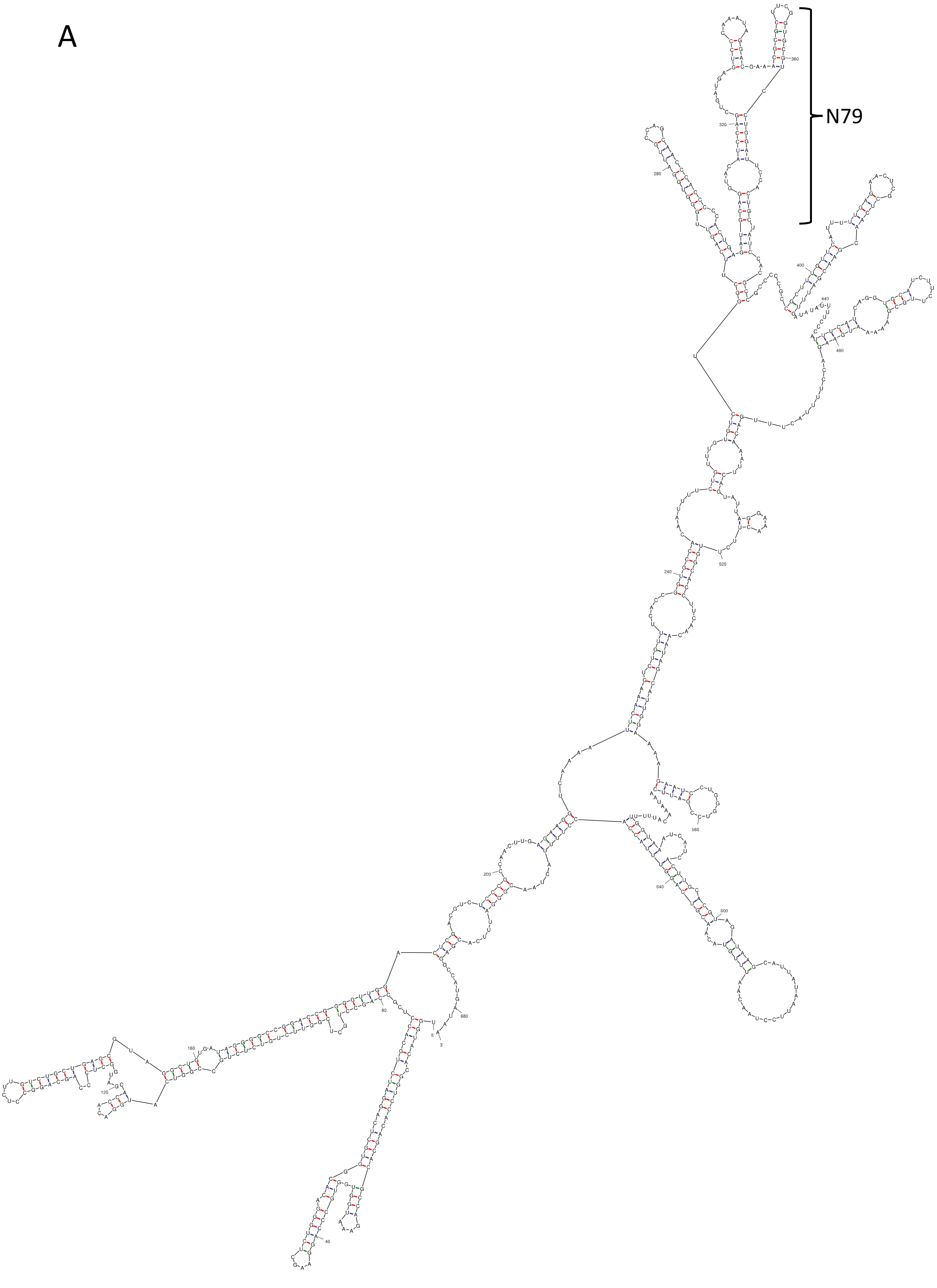

B

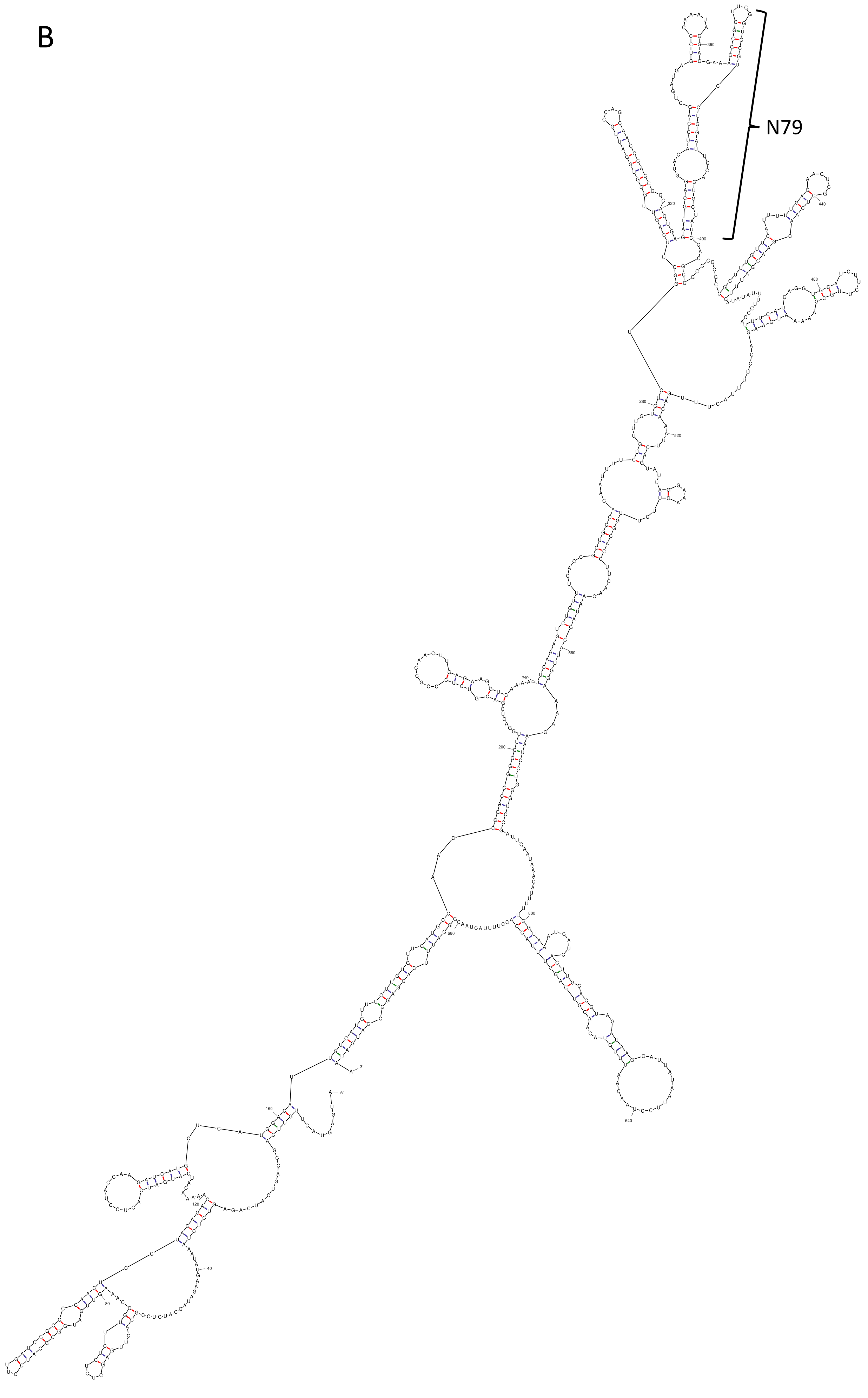

C

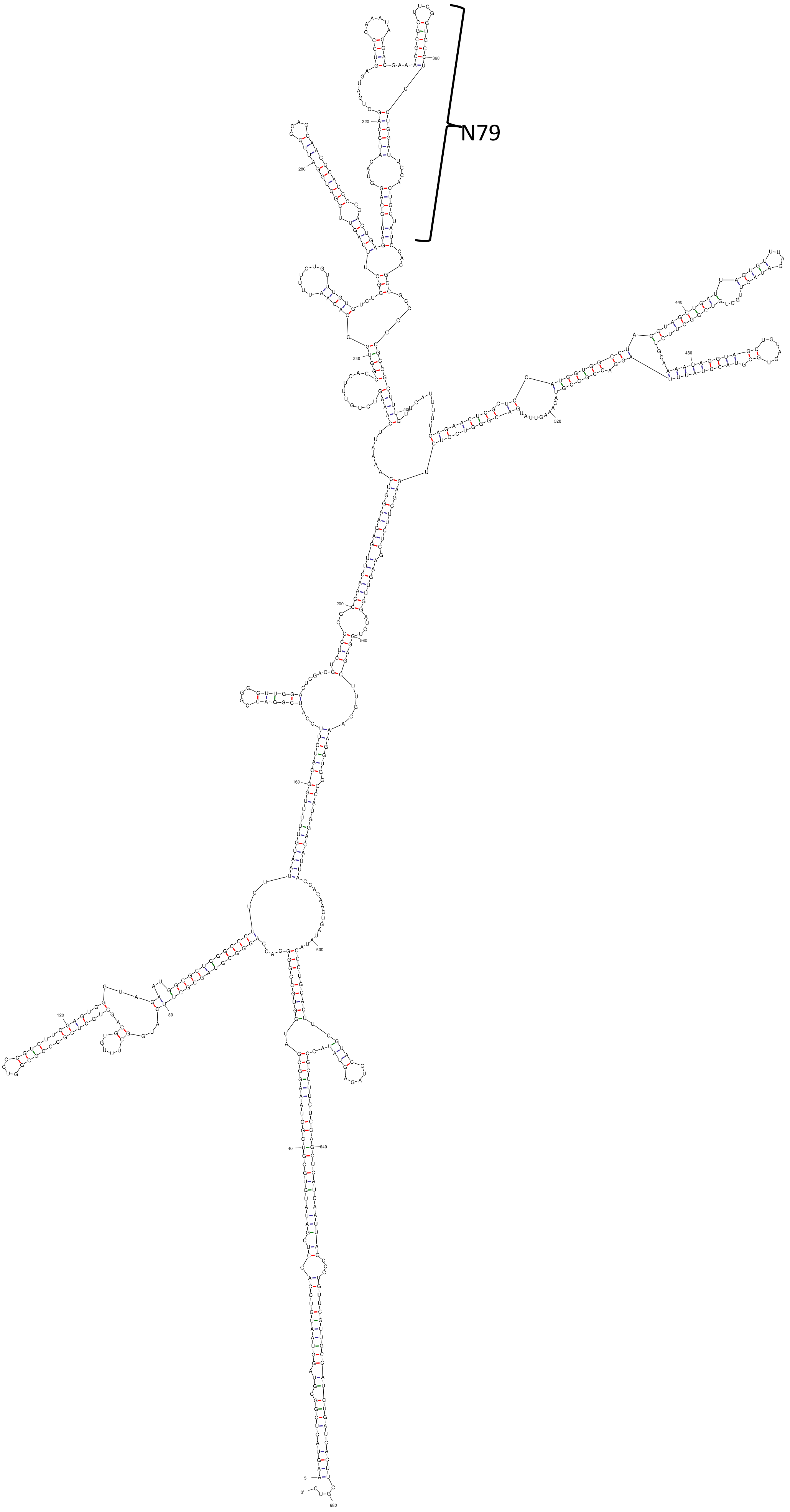

D

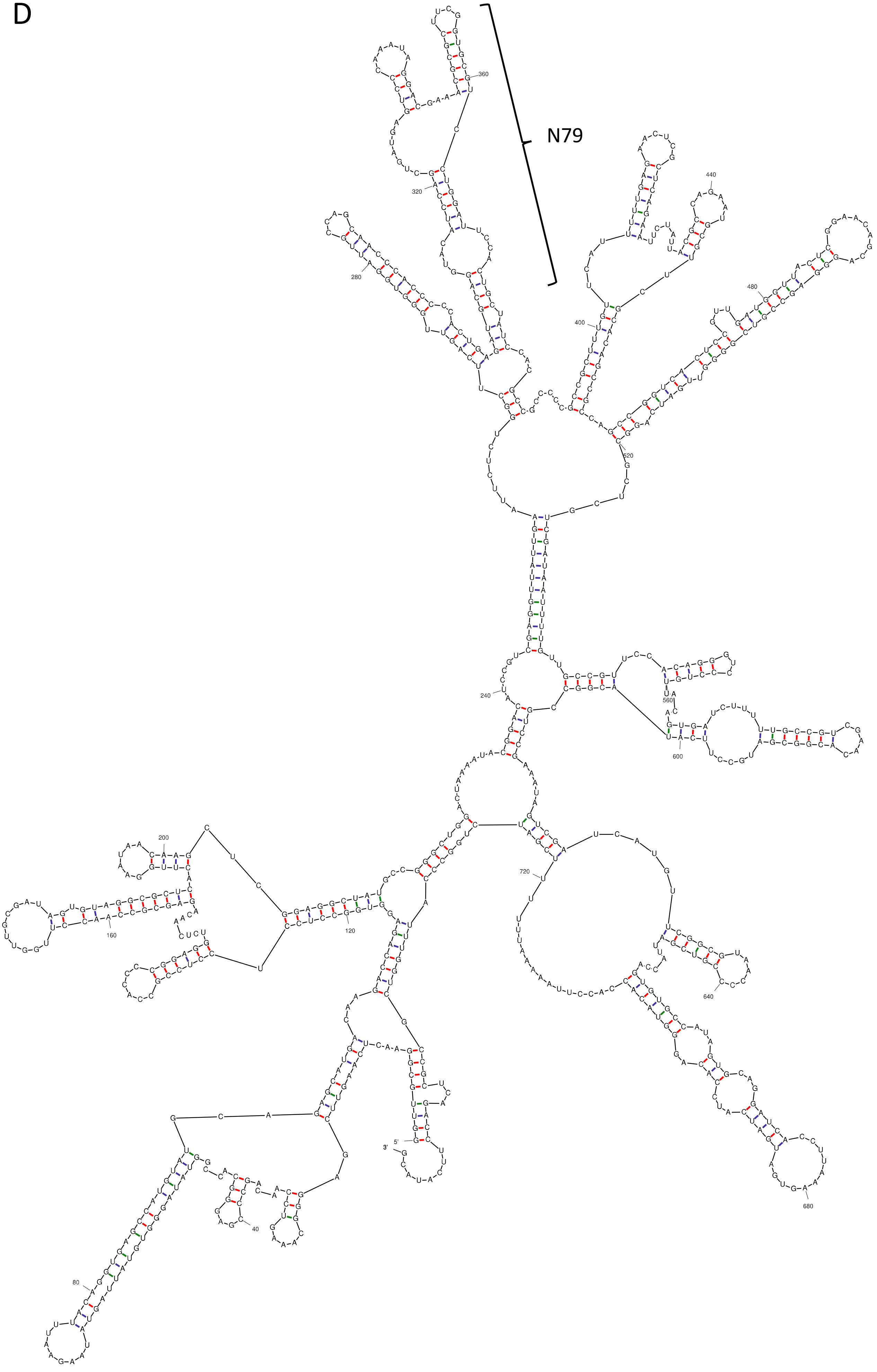

E

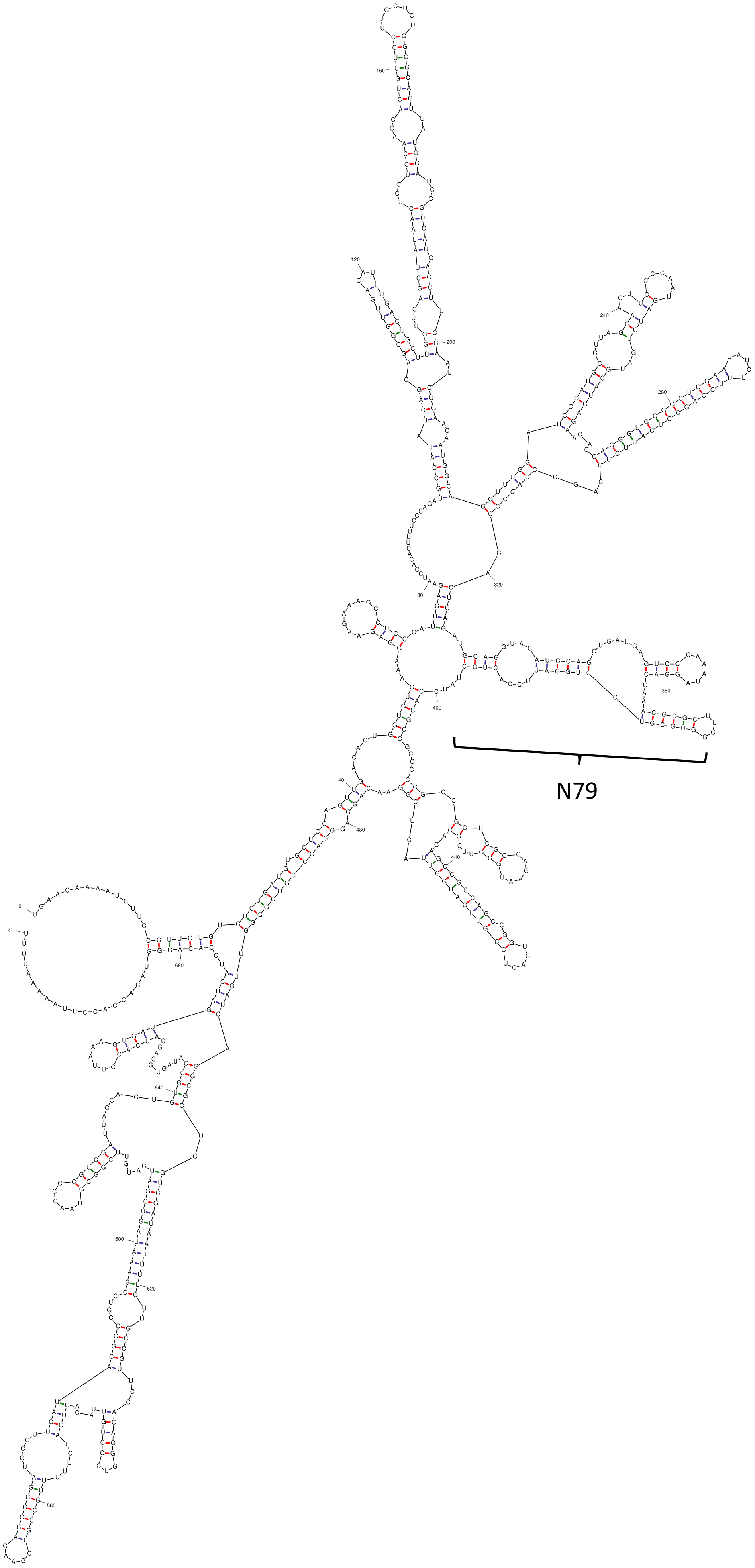

F

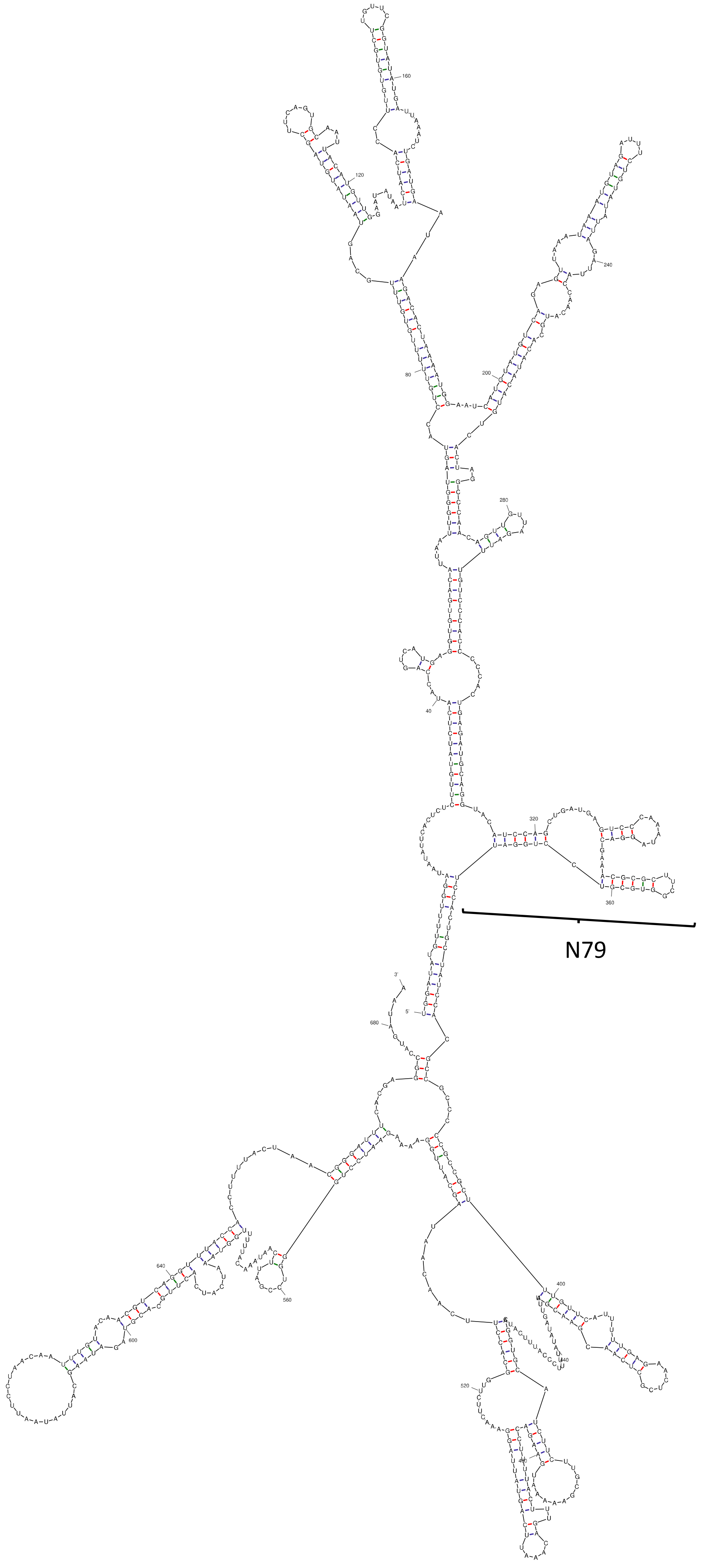

Fig S2. For each viral construct examined, the N79 ribozyme RNA folding structure was modelled in the context of 300 nts of upstream and downstream negative complimentary sequence (panels A, B, C, D, E and F represent HCV, YFV, CHIKV, HEV, FCV and HRV respectively). All RNA models were produced by *in silico* free energy minimisation using the UNAFOLD package with default settings.
