# Supplementary material for: Positive strand RNA viruses differ in the constraints they place on the folding of their negative strand
Source: RNA. 2022 Oct;28(10):1359–76. doi: 10.1261/rna.079125.122 (PMC9479745; doi:10.1261/rna.079125.122)
Supplement: Supplemental Material [file supp_079125.122_Supplemental_Fig_S3.pdf]

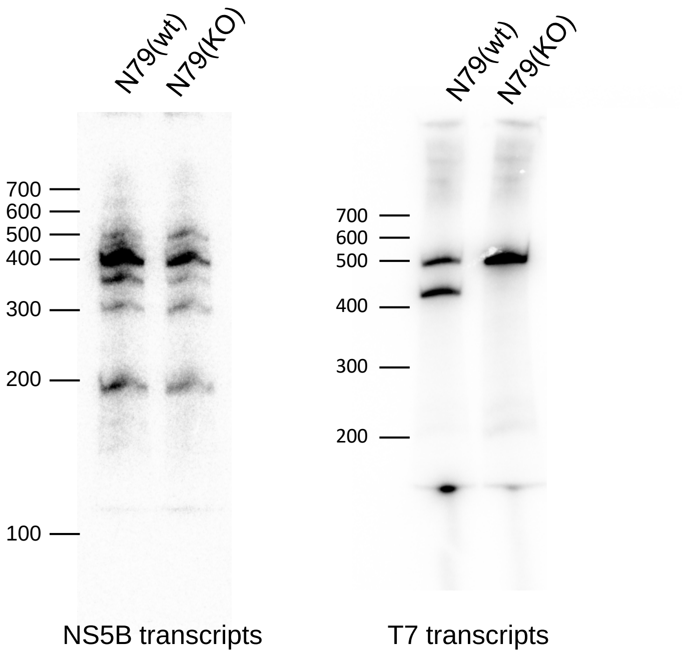

Fig S3 Gel analysis of N79 Rbz containing RNAs transcribed by NS5B and T7 polymerases in reactions incubated at 37°C.
